# Supplementary material for: Adenoviral Vector Vaccination Induces a Conserved Program of CD8+ T Cell Memory Differentiation in Mouse and Man
Source: Cell Rep. 2015 Nov 12;13(8):1578–88. doi: 10.1016/j.celrep.2015.10.034 (PMC4670868; doi:10.1016/j.celrep.2015.10.034)
Supplement: Document S1. Supplemental Experimental Procedures and Figures S1–S5 [file mmc1.pdf]

Cell Reports

Supplemental Information

Adenoviral Vector Vaccination Induces a Conserved Program of CD8$^+$ T Cell Memory Differentiation in Mouse and Man

Beatrice Bolinger, Stuart Sims,, Leo Swadling, Geraldine O’Hara, Catherine de Lara, Dilair Baban, Natasha Saghal, Lian Ni Lee, Emanuele Marchi, Mark Davis, Evan Newell, Stefania Capone, Antonella Folgori, Ellie Barnes, and Paul Klenerman

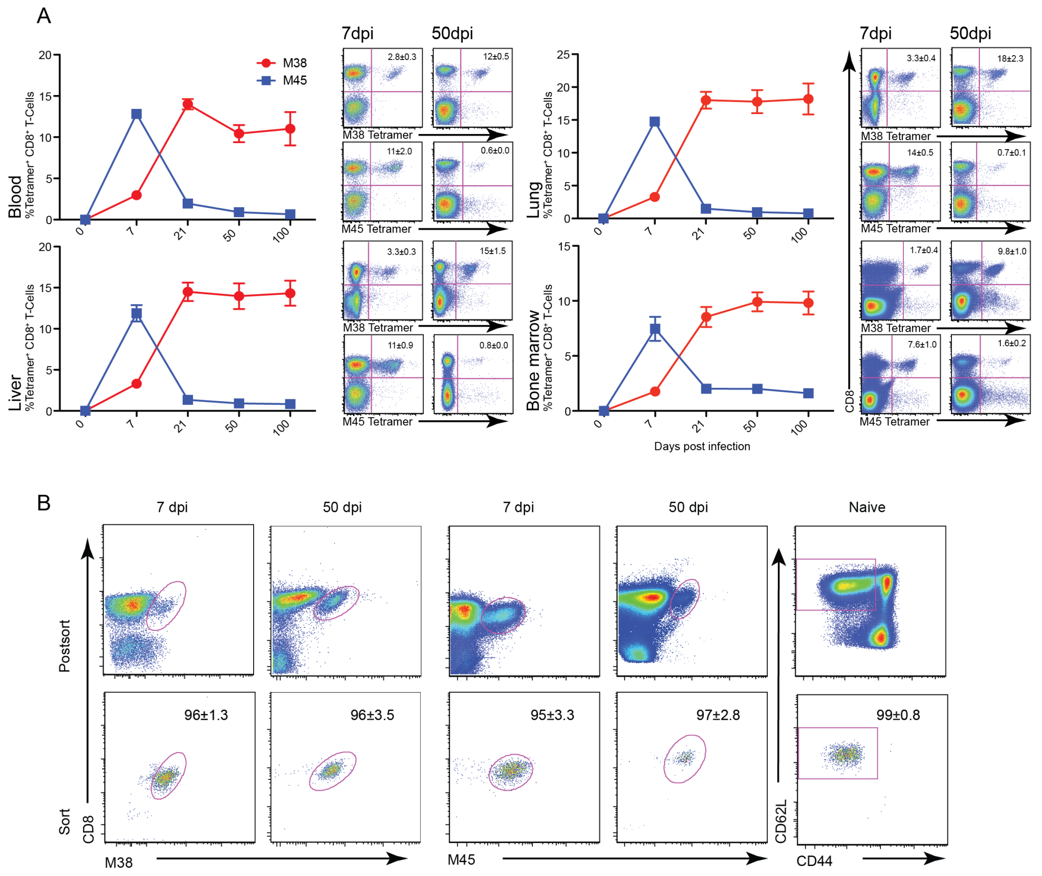

Figure S1: MCMV-specific inflating CD8 T cells are not restricted to the spleen (related to Figure 1).

C57BL/6 mice were infected i.v. with $1x10^6$ pfu MCMV.

(A) Longitudinal flow cytometry analysis showing the percentage of M38- (red) and M45- (blue) specific $CD8^+$ T cells in blood, liver, lung and bone marrow at 0, 7, 21, 50 and 100 days post infection. Mean percentage of live tetramer-positive $CD8^+$ lymphocytes is indicated (n=8, mean±SEM). Representative flow cytometry plots of M38- and M45-specific $CD8^+$ T cells 7 and 50 days post infection in blood, liver, lung and bone marrow.

(B) Representative flow cytometry plots from cell sorts of M38- and M45-specific $CD8^+$ T cells 7 and 50 days post infection. Cell sorts were performed in triplicates; five mice were pooled per group. Mean percentage of live tetramer-positive $CD8^+$ lymphocytes is indicated (n=3, mean±SEM).

A

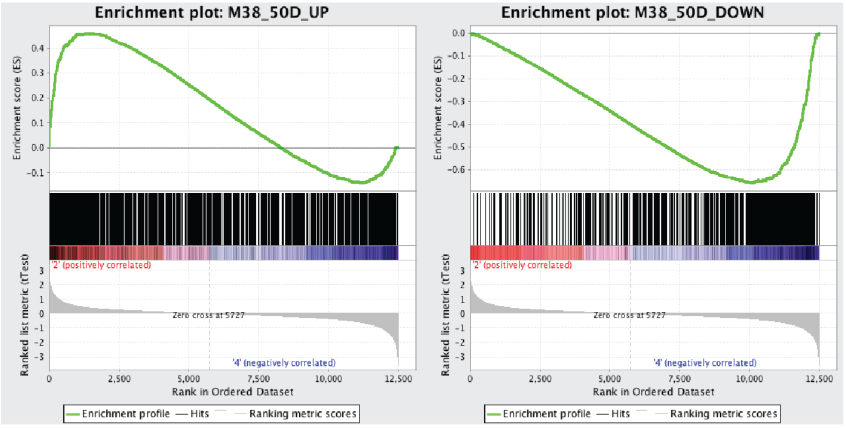

B

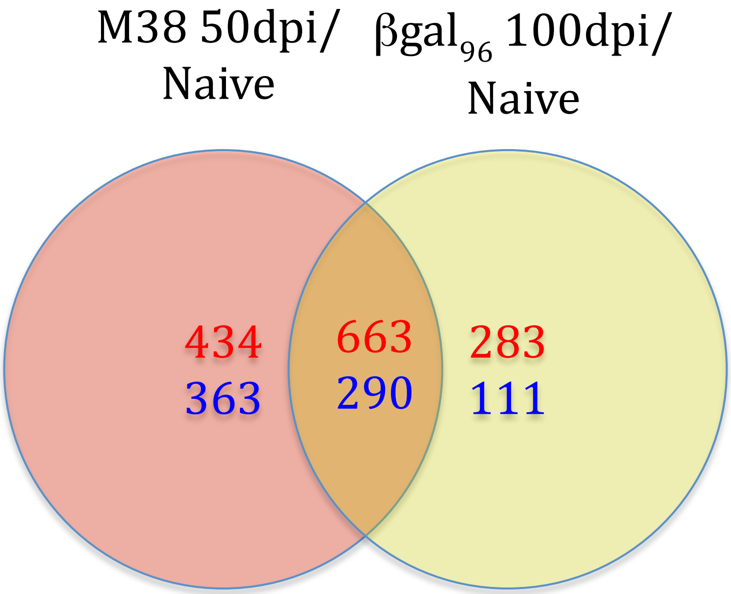

Figure S2: MCMV- and βgal-specific expanded CD8 T cells display similarities in the gene expression profile (related to Figure 2).

(A) Gene set enrichment analysis (GSEA) of genes of sustained MCMV-specific and Ad-LacZ-specific $CD8^+$ T cells. (B) Venn diagram showing the number of differentially expressed genes between βgal_{96}- specific $CD8^+$ T cells 100 (yellow) days post immunization and M38-specific $CD8^+$ T cells 50 (red) days post infection. Filter criteria of at least 2 fold change with $P \leq 0.05$

compared to naïve $CD8^+$ T cells. Up-regulated genes are indicated in red and down-regulated genes in blue.

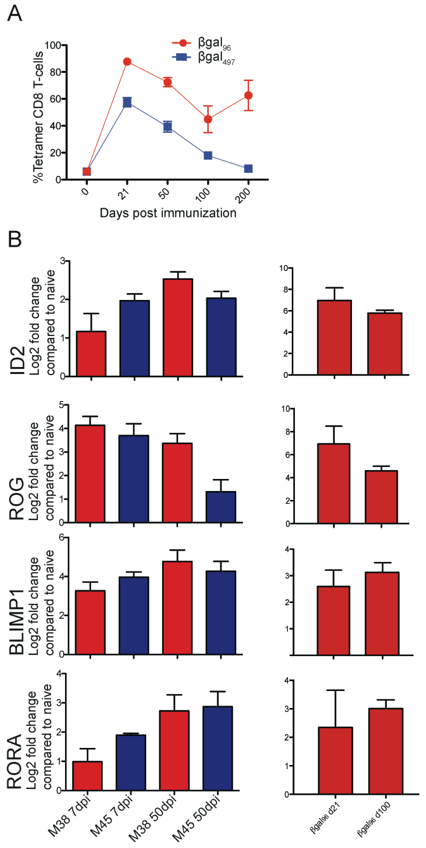

Figure S3: Tbet on βgal-specific CD8⁺ T cell; mRNA expression of the transcriptional regulators ID2, ROG, BLIMP1 and RORA (related to Figure 4).

(A) Flow cytometry analysis showing the percentage of $CD8^+$ T cells expressing the transcription factor Tbet on βgal_{96}- (red) and βgal_{497}-specific (blue) $CD8^+$ T cells in the spleen (n=5-9, ±SEM).

(B) ID2, ROG, BLIMP1 and RORA mRNA expression was determined by quantitative RT-PCR in M38- and M45-specific $CD8^+$ T cells at day 7 and 50 post infection and in βgal_{96}-specific $CD8^+$ T cells d21 and d100 after immunization. Five mice were pooled for each group; real-time analysis, performed in triplicates. Data presented as Log2 fold change compared to naive $CD8^+$ T-cells after normalization against the internal control gene HPRT.

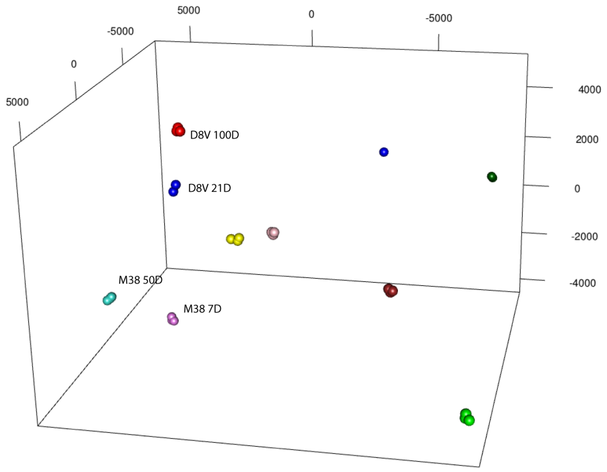
D8V 100D
D8V 21D
M38 50D
M38 7D

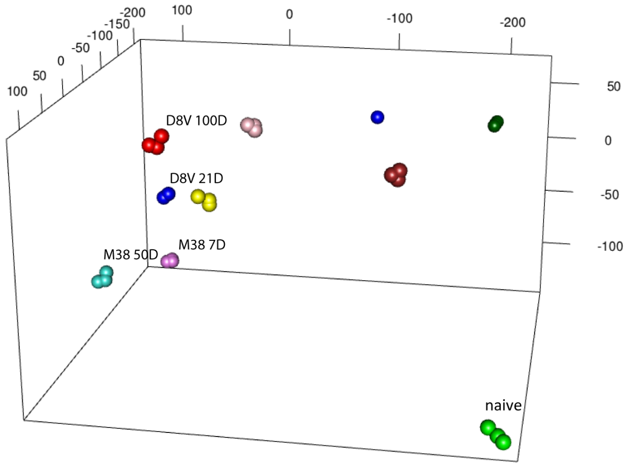
D8V 100D
D8V 21D
M38 50D
M38 7D
naive

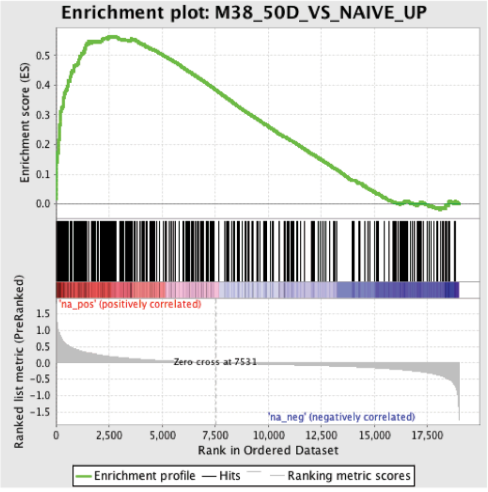
Enrichment plot: M38_50D_VS_NAIVE_UP
Enrichment score (ES)
Ranked list metric (PreRanked)
'na_pos' (positively correlated)
Zero cross at 7531
'na_neg' (negatively correlated)
Rank in Ordered Dataset
Enrichment profile — Hits — Ranking metric scores

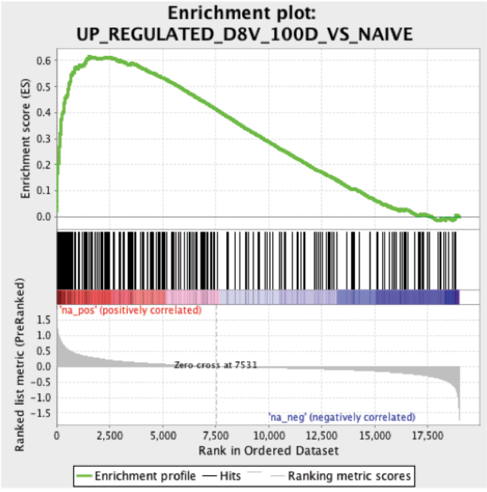
Enrichment plot:
UP_REGULATED_D8V_100D_VS_NAIVE
Enrichment score (ES)
Ranked list metric (PreRanked)
'na_pos' (positively correlated)
Zero cross at 7531
'na_neg' (negatively correlated)
Rank in Ordered Dataset
Enrichment profile — Hits — Ranking metric scores

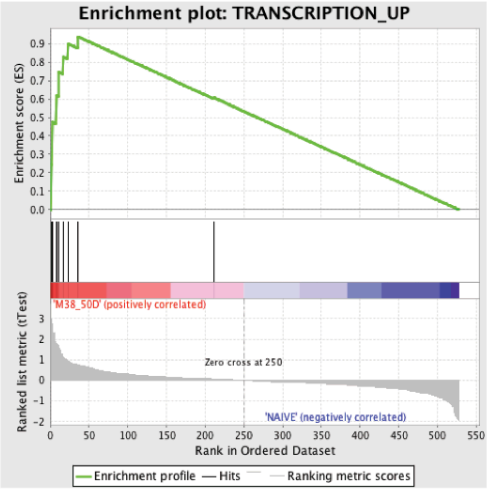
Enrichment plot: TRANSCRIPTION_UP
Enrichment score (ES)
Ranked list metric (tTest)
'M38_50D' (positively correlated)
Zero cross at 250
'NAIVE' (negatively correlated)
Rank in Ordered Dataset
Enrichment profile — Hits — Ranking metric scores

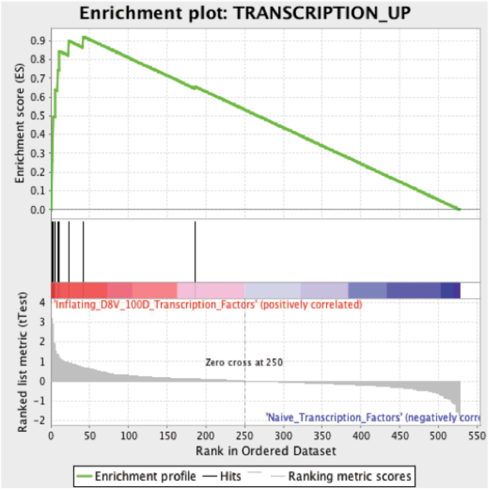
Enrichment plot: TRANSCRIPTION_UP
Enrichment score (ES)
Ranked list metric (tTest)
'Inflating_D8V_100D_Transcription_Factors' (positively correlated)
Zero cross at 250
Rank in Ordered Dataset
Enrichment profile — Hits — Ranking metric scores

Figure S4: Principal Components Analysis (PCA) and Gene set enrichment analysis (GSEA) of differential regulated genes of inflationary MCMV-specific or Ad-LacZ-specific CD8+ T cells (related to Figure 4).

Upper panels: Left hand panel shows 3D PCA of all microarray samples employing the entire set of probe intensities (following removal only of controls and weakly detected probes, 17062 total probes). The transcription profiles plotted are: naïve (green), bgal96-specific d21 (blue), bgal96-specific d100 (red), bgal497-specific d21 (yellow), bgal497-specific d100 (dark green), M38-specific d7 (violet), M38-specific d50 (turquoise), M45-specific d7 (pink) and M45-specific CD8+ T cells d50 (brown). Right hand panel is as above but PCA performed using only the subset of significantly differentially modulated genes defined as TFs (see methods).

Lower panels: GSEA was performed as indicated in the methods. The plots indicate strong, statistically robust enrichment for both up and down-regulated genes (GSEA for down-regulated genes not shown) shared between the two datasets. Left panels GSEA of genes of M38-specific and human CMV-specific inflationary CD8+ T cells, right panels GSEA of genes of bgal96-specific and human CMV-specific inflationary CD8+ T cells (Hertoghs et al., 2010). Lower panels show the same analyses using TFs only.

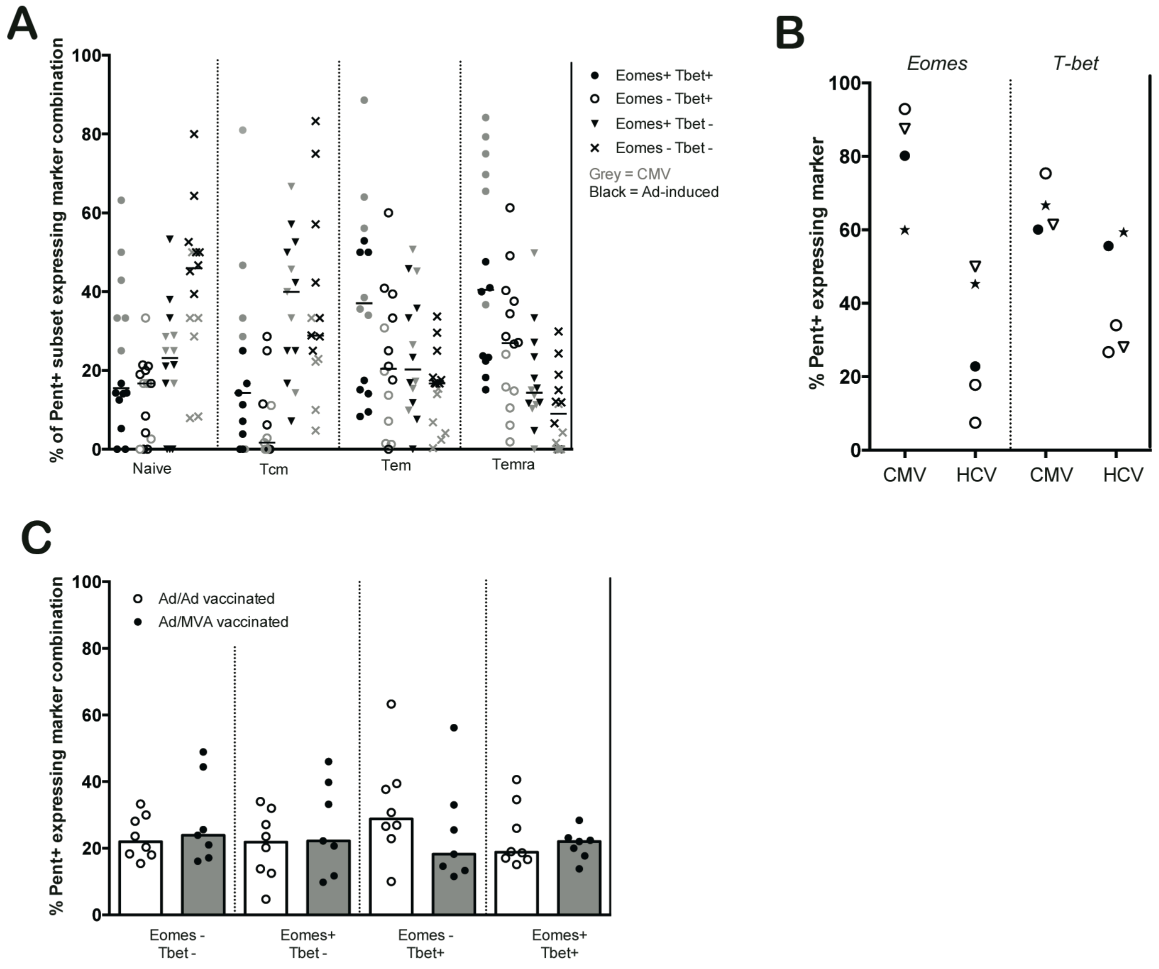

Figure S5: Eomes and Tbet expression in antigen-specific T cells (related to Figure 5): (A) HCV-specific T memory cells induced by virally vectored vaccine regimes (black; 8-26 weeks post boost vaccination) or by natural infection with CMV (grey) were identified by pentamer staining and split into T cell memory subsets using CD45RA and CCR7 staining (Naïve: CD45RA+CCR7+; Tcm: CD45RA-CCR7+; Tem: CD45RA-CCR7-; Temra: CD45RA+CCR7-). The co-expression of T-bet and Eomes was assessed on each T cell subset (CMV n=8; Ad/Ad vaccination n=8, Ad/MVA n=6). Line at median. (B) In four vaccinated individuals CMV-specific memory T cells could be detected and the expression pattern of Eomes and Tbet is shown in both vaccine induced HCV-specific T cells and CMV-specific T cells. **(C)** A comparison of the co-expression of T-bet and Eomes on vaccine induced T cells in individuals who received either ChAd3/Ad6 or ChAd3-MVA heterologous prime-boost vaccination regimes. Bars at median.

Supplementary Experimental Procedures

Intracellular staining and peptide stimulation

Peptide stimulation: $1x10^6$ cells were stimulated for 2 hours at 37°C with either 10^{-4} M M38 or 10^{-4}M M45 peptide. As a positive control cells were stimulated with phorbol myristate acetate (PMA) (50ng/ml) and ionomycin (500ng/ml) or left untreated as a negative control. After 2 hours GolgiPlug (1μl/1ml final concentration) from BD Bioscience (Oxford, UK) was added to each well and cells incubated for a further 4 hours at 37°C. Cells were fixed with the cytofix/cytoperm solution from BD Bioscience (Oxford, UK) and stained and washed with Perm/Wash buffer from BD Bioscience (Oxford, UK).

Microarray data analysis

Gene expression data were obtained by hybridising a total of 12 samples from 4 experimental groups (n=3 per group) to Illumina MouseWG6 Expression BeadChips. Raw data were exported from the Illumina GenomeStudio software (v1.0.6) for further processing and analysis using R statistical software (v2.10) and BioConductor packages. Raw signal intensities were background corrected using array-specific measures of background intensity based on negative control probes, prior to being transformed and normalized using the 'vsn' package. Quality control analyses did not reveal any outlier samples. The dataset was then filtered to remove probes not detected (detection score <0.95) in any of the samples.

Statistical analysis was performed using the Linear Models for Microarray Analysis (limma) package. Differential expression between the experimental groups was assessed by generating relevant contrasts corresponding to the various two-group comparisons. Raw p-values were corrected for multiple testing using the false discovery rate controlling procedure of Benjamini and Hochberg, adjusted p-values below 0.01 were considered significant. Significant probe lists were then annotated using the relevant annotation file (MouseWG6_V2_0_R0_11278593_A) that was downloaded from the Illumina website (http://www.illumina.com) for further biological

investigation. To compare gene lists, these were ranked according to the t statistics or by log fold change and gene set enrichment analysis (GSEA) was run (www.broadinstitute.org/gsea/index.jsp). PCA of whole expression profiles was performed using R language and MeV software (http://www.tm4.org) using all genes probes, and repeated using the subset of significantly expressed transcription factors according ANOVA test across all samples groups.

Pentamer staining and flow cytometry

Fluorescence minus one (FMO) samples and isotype controls were performed using a CMV pentamer on CMV^{+} PBMCs, and fixed gating strategies were used throughout. The specificity of pentamers was tested on HLA-matched pre-vaccination samples from healthy volunteers (Barnes et al., 2012). PE-labeled class I pentamers loaded with HCV $NS3_{1406\text{-}1415}$ (KLSALGINAV), HCV $NS3_{1073\text{-}1081}$ (ATDALMTGY) CMV $pp65_{495\text{-}504}$ (NLVPMVATV), or FLU $M1/MP_{58–66}$ (GILGFVFTL) were obtained from ProImmune. The cells were co-stained with combinations of the following antibodies: CD3-PO (Invitrogen), CD8-Alexa700 (Biolegend), CCR7–PE-Cy7 (BD), CD45RA-FITC (BD; fluorescein isothiocyanate), T-bet-BV605 (Biolegend), Eomes-eFluor660 (eBiosciences).

PCA with CyTOF data

For PCA, cells were gated on live $CD3^{+}CD8^{+}$ T cells (principal component loaded using data from patient 319 at TW22 and then applied to the other data sets), and these events were exported to a tab-delimited text file with FlowJo v9.3.2 for further analysis with scripts written in MATLAB. FCS files containing these additional parameters were created using a custom algorithm written in Java (text to FCS script provided by W. Moore); pdb files were also created using MATLAB that could be read by PyMOL software (DeLano Scientific LLC). All MATLAB scripts started with transformation of data into logical biexponential scaling as described (Newell et al., 2012).

Cell preparation from bone marrow, lung and liver

Bone marrow was isolated by washing the femur shaft with PBS. Cells were passed through a 70µm nylon filter (BD) and red cell lysis was performed. Perfused livers were passed through a 70µm nylon filter (BD) and lymphocytes were purified by a Percoll (GE healthcare) gradient centrifugation. Lungs were minced with razor blades and incubated in PBS containing 60U/ml DNase (AppliChem) and 170U/ml collagenase II (Gibco) at 37°C for 45 min. Cell aggregates were dispersed by passing the digest through a 70µm nylon filter (BD). Absolute cell counts were determined by counting leukocytes in an improved Neubauer chamber.

RNA extraction and cDNA generation

Tetramer-sorted cells were resuspended in Trizol (Sigma-Aldrich, USA) and RNA was isolated by isopropanol precipitation, washed with ethanol 70% and resuspended in DEPC-water. RNA was DNAse treated with DNAse I (Invitrogen, Paisley, UK) and subjected to RT-PCR using 100ng purified RNA. For RT-PCR the high capacity cDNA archive Kit from Applied Biosystem (ABI PRISM, Warrington, United Kingdom) was used according to the specifications of the manufacturer.

Quantitative realtime PCR for selected genes

Quantitative real-time PCR was performed using a Light cycler 480 Real-Time PCR System (Roche Diagnostics) and the LightCycler 480 probes master reaction mix (Roche Diagnostics) following the manufacturer's protocol. Data analysis was performed with LightCycler 480 Software (Roche Diagnostics). Oligonucleotides were purchased from Eurofins MWG Operon (Ebersberg, Germany). Oligonucleotides sequences used as primers for quantitative real-time PCR and corresponding probes were designed according to the guidance of the Universal Probelibrary from Roche applied Science. Thermal cycling started with HotStarTaq activation during 10 min at 95°C. Thereafter 45 cycles of amplification were run consisting of 10 s at 95°C, 30 s 60°C, and 20 s of 72°C. A negative control, containing reagents only, and serial dilutions of cDNA were included in

each run. Each sample was measured as a triplicate and the average concentration was used. For LightCycler analysis, expression of hypoxanthine phosphoribosyltransferase gene (HPRT) was used for normalization. Relative expression of samples from naive and MCMV-infected or Ad-LacZ immunized mice was calculated by the comparative cycling threshold method ($\Delta\Delta C_T$).

Barnes, E., Folgori, A., Capone, S., Swadling, L., Aston, S., Kurioka, A., Meyer, J., Huddart, R., Smith, K., Townsend, R., *et al.* (2012). Novel adenovirus-based vaccines induce broad and sustained T cell responses to HCV in man. Sci Transl Med *4*, 115ra111.

Hertoghs, K.M., Moerland, P.D., van Stijn, A., Remmerswaal, E.B., Yong, S.L., van de Berg, P.J., van Ham, S.M., Baas, F., ten Berge, I.J., and van Lier, R.A. (2010). Molecular profiling of cytomegalovirus-induced human CD8+ T cell differentiation. The Journal of clinical investigation *120*, 4077-4090.

Newell, E.W., Sigal, N., Bendall, S.C., Nolan, G.P., and Davis, M.M. (2012). Cytometry by time-of-flight shows combinatorial cytokine expression and virus-specific cell niches within a continuum of CD8+ T cell phenotypes. Immunity *36*, 142-152.
