# Supplementary material for: Adenoviral Vector Vaccination Induces a Conserved Program of CD8+ T Cell Memory Differentiation in Mouse and Man
Source: Cell Rep. 2015 Nov 12;13(8):1578–88. doi: 10.1016/j.celrep.2015.10.034 (PMC4670868; doi:10.1016/j.celrep.2015.10.034)
Supplement: Document S2. Article plus Supplemental Information [file mmc2.pdf]

Cell Reports

Report

Adenoviral Vector Vaccination Induces a Conserved Program of CD8$^+$ T Cell Memory Differentiation in Mouse and Man

Graphical Abstract

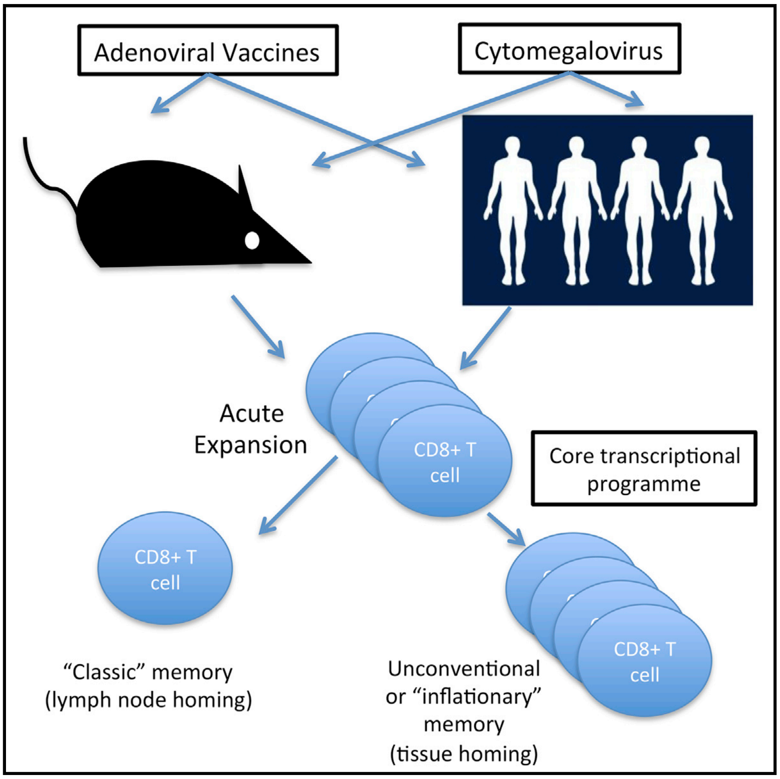

Highlights

- Adenovector vaccination induces two transcriptionally distinct CD8 memory responses
- The sustained response induced by adenovectors and CMV is closely related
- The core molecular features are shared tightly in mouse and man
- Adenovaccines in humans induce a CD8 response that recapitulates these core features

Authors

Beatrice Bolinger, Stuart Sims, Leo Swadling, ..., Antonella Folgori, Ellie Barnes, Paul Klenerman

Correspondence

beatrice.bolinger@unibas.ch

In Brief

Bolinger et al. define the transcriptional program associated with sustained CD8$^+$ T cell memory induced by adenoviral vaccination. They relate these to memory "inflation" induced following infection by persistent CMVs. Core features are found to be shared by mouse and man, including a prominent role for TBX21.

Bolinger et al., 2015, Cell Reports *13*, 1578–1588
November 24, 2015 ©2015 The Authors
http://dx.doi.org/10.1016/j.celrep.2015.10.034

OPEN ACCESS
CellPress

Cell Reports
Report

Adenoviral Vector Vaccination Induces a Conserved Program of CD8$^+$ T Cell Memory Differentiation in Mouse and Man

Beatrice Bolinger,[1,7,8,*] Stuart Sims,[1,8] Leo Swadling,[1] Geraldine O'Hara,[1] Catherine de Lara,[1] Dilair Baban,[2] Natasha Saghal,[2] Lian Ni Lee,[1] Emanuele Marchi,[1] Mark Davis,[3] Evan Newell,[4] Stefania Capone,[5] Antonella Folgori,[5] Ellie Barnes,[1,6] and Paul Klenerman[1,6]
[1]Peter Medawar Building for Pathogen Research, University of Oxford, Oxford OX1 3SY, UK
[2]Wellcome Trust Centre for Human Genetics, Roosevelt Drive, Oxford OX3 7BN, UK
[3]Department of Microbiology and Immunology, Stanford University, Stanford, CA 94305, USA
[4]Singapore Institute for Clinical Sciences, Agency of Science Technology and Research (A*STAR), Singapore 138632, Singapore
[5]ReiThera, Viale Città d'Europa 679, 00144 Roma, Italy
[6]NIHR Biomedical Research Centre, Oxford OX3 9DU, UK
[7]Department Biomedicine, University of Basel, 4056 Basel, Switzerland
[8]Co-first author
*Correspondence: beatrice.bolinger@unibas.ch
http://dx.doi.org/10.1016/j.celrep.2015.10.034
This is an open access article under the CC BY license (http://creativecommons.org/licenses/by/4.0/).

SUMMARY

Following exposure to vaccines, antigen-specific CD8$^+$ T cell responses develop as long-term memory pools. Vaccine strategies based on adenoviral vectors, e.g., those developed for HCV, are able to induce and sustain substantial CD8$^+$ T cell populations. How such populations evolve following vaccination remains to be defined at a transcriptional level. We addressed the transcriptional regulation of divergent CD8$^+$ T cell memory pools induced by an adenovector encoding a model antigen (beta-galactosidase). We observe transcriptional profiles that mimic those following infection with persistent pathogens, murine and human cytomegalovirus (CMV). Key transcriptional hallmarks include upregulation of homing receptors and anti-apoptotic pathways, driven by conserved networks of transcription factors, including T-bet. In humans, an adenovirus vaccine induced similar CMV-like phenotypes and transcription factor regulation. These data clarify the core features of CD8$^+$ T cell memory following vaccination with adenovectors and indicate a conserved pathway for memory development shared with persistent herpesviruses.

INTRODUCTION

After viral infection, naive antigen-specific CD8$^+$ T cells clonally expand and differentiate. Massive proliferation and differentiation to effector T cells is coupled to changes in homing, function, and gene expression, leading to CD8$^+$ T cell memory. Defining mechanisms that drive effective CD8$^+$ T cell memory pools is critical for the design of vaccines.

Broadly, two subsets of memory CD8$^+$ T cells are described: central memory CD8$^+$ T cells (T_{CM}) and the effector memory CD8$^+$ T cells (T_{EM}) (Sallusto et al., 1999). "Central" memory pools are typically contracted memory populations that express lymph-node-homing markers (CD62L and CCR7). "Effector memory" subsets lack these and are found distributed in tissues, e.g., lung and liver. These pools are linked to infection with persistent viruses, the best examples being human and murine cytomegaloviruses (HCMV and MCMV).

A characteristic of the CMV immunobiology is the induction of an expanded, sustained effector-memory T cell response to specific epitopes, a phenomenon termed CD8$^+$ T cell "memory inflation" (Karrer et al., 2003). In parallel, classic "non-inflating," central memory responses develop against many epitopes. Molecular profiling of CMV-specific CD8$^+$ T cells in humans revealed that the development of CMV-specific CD8$^+$ T cells is a dynamic process, with key features of the HCMV-specific CD8$^+$ T cell phenotype installed early after infection (Hertoghs et al., 2010).

CD8$^+$ T cell memory induced by vaccines could provide protection against complex pathogens. Whereas CMV-based vectors show promise in studies of SIV infection (Hansen et al., 2011), such viruses are complex. One technology that has shown potency in generation of antiviral T cell pools in clinical studies is based on replication-deficient adenoviral vectors. Several trials have indicated such vectors are safe and can induce substantial immune responses against pathogens such as HCV (Barnes et al., 2012; Colloca et al., 2012).

Studies of vaccine-induced T cell responses in a murine model using a recombinant replication-deficient HuAd5 vector expressing lacZ (Ad-lacZ) (Bolinger et al., 2013) revealed two distinct pathways for memory—an inflationary response to one epitope and a typical contracting response to a second epitope. The sustained response showed phenotypic features typical of effector memory and was enriched in tissues, whereas the reverse was true for the contracting response.

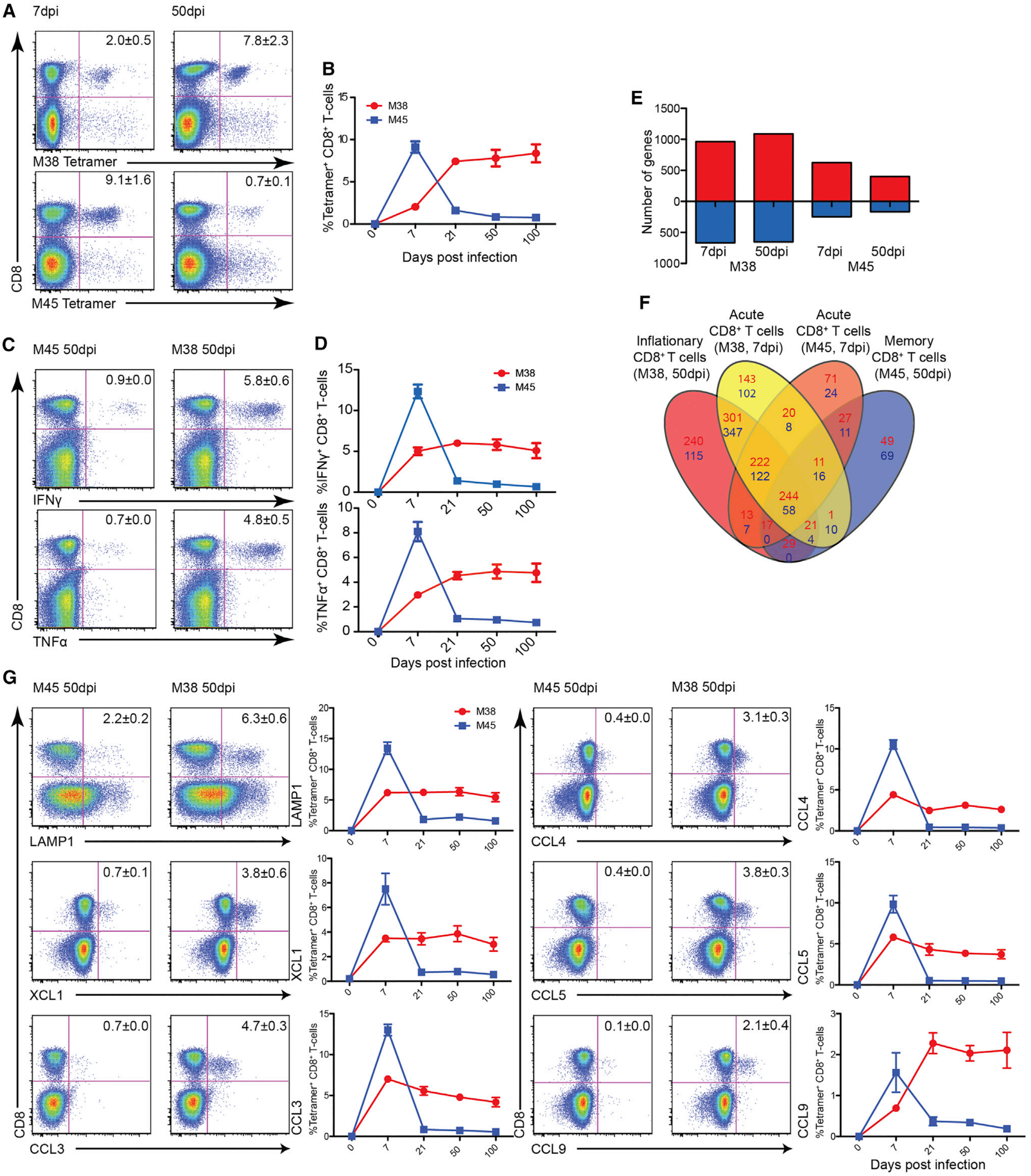

Figure 1. Frequency, Function, and Gene Expression Signature of MCMV-Specific CD8⁺ T Cells

C57BL/6 mice were infected intravenously (i.v.) with 1 × 10^6 pfus MCMV.

(A) Tetramer staining for M38- and M45-specific CD8⁺ T cells on 7 and 50 days postinfection in spleen. Mean percentages of live tetramer⁺ CD8⁺ T cells are indicated (n = 8; mean ± SEM).

(B) Time course for M38- (red) and M45- (blue) specific CD8⁺ T cells. Splenocytes from 0, 7, 21, 50, and 100 days postinfection mice were stained with tetramers and analyzed by flow cytometry. Mean percentages of live tetramer⁺ CD8⁺ lymphocytes are indicated (n = 8; mean ± SEM).

(legend continued on next page)

Because these peptide epitopes are both derived from the same expressed transgene, this model provides a controlled system for analysis of two divergent vaccine-induced memory pools.

Here, we define the transcriptional changes in MCMV infection and Ad-LacZ vaccination and addressed to what extent parallel changes can be observed in human memory pools induced by CMV and adenoviral vectors. Our data clearly show that a subset of stable memory CD8$^+$ T cell responses, whether induced by vaccine vectors or natural virus, in mouse and man, display a common molecular profile divergent from that of acute effector, central memory, and exhausted CD8$^+$ T cells.

RESULTS

Two Functional and Transcriptionally Distinct Memory Patterns of MCMV-Specific CD8$^+$ T Cells

To establish a data set for gene expression in CD8$^+$ T cell memory pools in the setting of a persistent infection, we analyzed the well-characterized model of MCMV infection. This has the advantage of a parallel human data set for comparison (Hertoghs et al., 2010). Infection of C57BL/6 mice with MCMV resulted in two distinct CD8$^+$ T cell responses, the conventional (non-inflationary) and the expanded (inflationary) CD8$^+$ T cell response in blood, spleen, and organs (liver or lung; Figures 1A, 1B; Figure S1A). An analogous profile was observed when M45- and M38-specific CD8$^+$ T cells were analyzed for IFNγ and TNFα production (Figures 1C and 1D). Furthermore, the expression of chemokines XCL1, CCL3, CCL4, CCL5, and CCL9 (Figure 1G) and LAMP1 followed the same pattern. These results confirm previous data that, after MCMV infection, two distinct types of CD8$^+$ T cell responses are induced and demonstrate that both types of CD8$^+$ T cells retained polyfunctionality over time (Munks et al., 2006; Sierro et al., 2005).

To generate gene-expression profiles of MCMV-specific memory and effector CD8$^+$ T cells, M38- and M45-specific CD8$^+$ T cells from days 7 and 50 postinfection were tetramer sorted. As naive controls, sorted CD62L^{hi} CD44^{lo} CD8$^+$ T cells from naive C57BL/6 mice were used (Figure S1B). We compared gene expression in M38- and M45-specific CD8$^+$ T cells from day 7 and day 50 to naive CD8$^+$ T cells. Considering genes which were >2-fold differentially expressed over naive CD8$^+$ T cells (adjusted $p < 0.01$) revealed a total of 627 upregulated and 246 downregulated genes for the M45-specific population on day 7 postinfection and 400 up- and 168 downregulated genes on day 50 postinfection. For the early M38-specific population, a total of 964 genes were upregulated and 667 genes downregulated, rising to 1,088 up- and 553 downregulated genes for the late, inflating M38-specific CD8$^+$ T cells (Figure 1E; GEO: GSE73314).

Comparison showed that the expression profiles of the four populations differed markedly (Figure 1F). M45-specific CD8$^+$ T cells downregulated a large number of genes from the acute phase as they entered the central memory phase and most closely resembled naive CD8$^+$ T cells. In contrast, M38-specific CD8$^+$ T cells maintained expression of many of their acute phase genes (1,324 shared genes), also acquiring inflation-specific genes, totaling overall the highest number of differentially expressed genes (Figure 1F). These data confirm that MCMV, on the transcriptional level, clearly induced two distinct CD8$^+$ T cell memory responses maintained in parallel.

Inflationary CD8$^+$ T Cell Memory Induced by a Non-replicating Adenovirus Vector

We next addressed the qualities of the vaccine-induced responses following administration of a replication-deficient βgal-recombinant adenovirus vector (Ad-LacZ) (Bolinger et al., 2013). Intravenous (i.v.) immunization of C57BL/6 mice with Ad-LacZ induced two types of CD8$^+$ T cell responses to βgal. βgal$_{96}$- and βgal$_{497}$-specific CD8$^+$ T cells displayed similar expansion on day 21 postimmunization, but at day 50, βgal$_{96}$-specific CD8$^+$ T cells had further increased to 13% of total CD8$^+$ T cells, whereas βgal$_{497}$-specific CD8$^+$ T cells contracted (Figures 2A and 2B). Consistent with previous studies (Bolinger et al., 2013), these responses maintained functionality and were enriched in tissues such as lung and liver (data not shown).

To examine further the function and stability of vaccine-induced responses, we used an adoptive transfer approach. Cells from CD45.1$^+$ mice were transferred at different time points after vaccination to CD45.2$^+$-vaccinated or naive mice and tracked using tetramers. The extended CD8$^+$ T cell responses were sustained over 8 weeks in the recipients, even in the absence of antigen, maintaining their phenotype (CD44^{hi}, CD62L^{lo}, CD27^{lo}, and CD127^{lo}) and distributing to tissues such as liver and lung (data not shown). Overall, these features are consistent with those of a stable memory T cell pool.

To assess the T cell transcriptome of βgal-specific CD8$^+$ T cells after Ad-LacZ immunization, a gene expression array analysis of tetramer-sorted βgal-specific CD8$^+$ T cells from day 21 and day 100 and of naive CD8$^+$ T cells (CD62L^{hi} and CD44^{lo}) was

(C) Representative flow cytometry plots of splenocytes producing IFNγ or TNFα stimulated with either M38 or M45 peptide in mice 7 and 50 days postinfection, gated on live lymphocytes. Numbers indicate the percentage of IFNγ- and TNFα-positive CD8$^+$ T cells (n = 8; mean ± SEM).

(D) Time course plotting the percentage of M38- (red) or M45- (blue) specific CD8$^+$ T cells from the spleen producing IFNγ and TNFα after stimulation with either the M38 or M45 peptide. Mean percentage of IFNγ and TNFα producing cells within the CD8$^+$ T cell compartment is indicated (n = 8; mean ± SEM).

(E) Number of genes upregulated (red) and downregulated (blue) in M38- and M45-specific CD8$^+$ T cells 7 and 50 days postinfection, compared to naive CD8$^+$ T cells. Filter criteria of at least 2-fold change with $p \leq 0.05$ compared to naive CD8$^+$ T cells are shown.

(F) Venn diagram showing the number of differentially expressed genes between M38-specific CD8$^+$ T cells 7 (yellow) and 50 (red) days postinfection and M45-specific CD8$^+$ T cells 7 (orange) and 50 (blue) days postinfection. Filter criteria of at least 2-fold change with $p \leq 0.05$ compared to naive CD8$^+$ T cells are shown. Upregulated genes are indicated in red and downregulated genes in blue.

(G) Representative flow cytometry plots of splenocytes expressing LAMP1 or producing effector molecules stimulated with either M38 or M45 peptide in mice days 7 and 50 postinfection, gated on live lymphocytes (n = 8; ±SEM). Longitudinal flow cytometry analysis shows the percentage of M38- (red) or M45- (blue) specific CD8$^+$ T cells from the spleen producing effector molecules after stimulation with either the M38 or M45 peptide. Mean percentages of LAMP1-, XCL1-, CCL3-, CCL4-, CCL5-, and CCL9-positive cells within the CD8$^+$ T cell compartment are indicated (n = 8; ±SEM).

See also Figure S1.

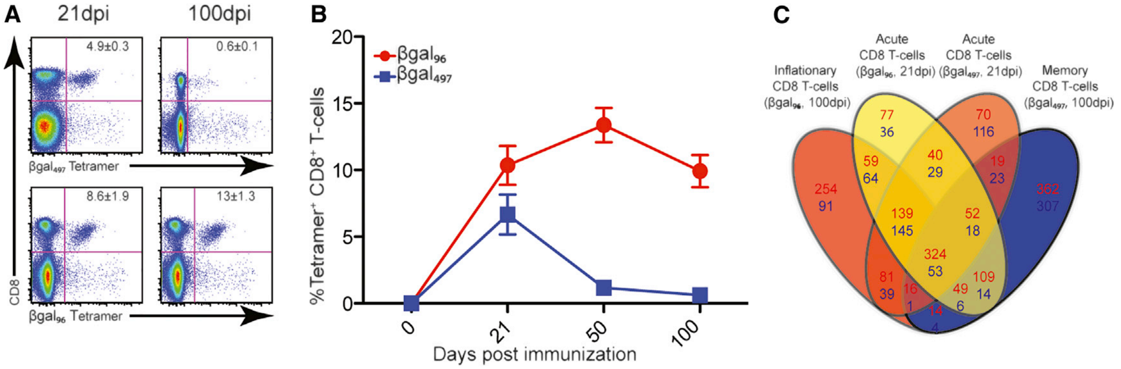

Figure 2. Frequency and Gene Expression Signature of Vaccine-Induced βgal-Specific CD8+ T Cells

C57BL/6 mice were immunized i.v. with 1×10^9 pfus Ad-LacZ.

(A) Representative flow cytometry plots of tetramer staining for βgal_{96}- and βgal_{497}-specific $CD8^+$ T cells on 21 and 100 days postimmunization in the spleen. Mean percentage of live tetramer-positive $CD8^+$ T cells is indicated (n = 10; mean ± SEM).

(B) Time course for βgal_{96}- (red) and βgal_{497}- (blue) specific $CD8^+$ T cells. Splenocytes from 0, 21, 50, and 100 days postimmunization were stained with tetramers and analyzed by flow cytometry. Mean percentages of live tetramer-positive $CD8^+$ T cells are indicated (n = 5; mean ± SEM).

(C) Venn diagram showing the number of differentially expressed genes between βgal_{96}-specific $CD8^+$ T cells 21 (yellow) and 100 (red) days postimmunization and βgal_{497}-specific $CD8^+$ T cells 21 (orange) and 100 (blue) days postimmunization. Filter criteria of at least 2-fold change with $p \leq 0.05$ compared to naive $CD8^+$ T cells are shown. Upregulated genes are indicated in red and downregulated genes in blue.

See also Figure S2.

performed. By only considering genes that were at least 2-fold differentially expressed (adjusted $p < 0.01$) compared to naive $CD8^+$ T cells, we found a total of 937 up- and 403 downregulated genes for βgal_{96}-specific (expanded) $CD8^+$ T cells (Figure 2C). By directly comparing the βgal_{96}-specific $CD8^+$ T cell population with the βgal_{497}-specific memory population, it is evident that memory βgal_{96}-specific $CD8^+$ T cells showed a distinct expression profile—neither identical to effector $CD8^+$ T cells nor memory βgal_{497}-specific $CD8^+$ T cells (Figure 2C). These data confirm previous analyses indicating that two distinct memory pools are induced in parallel following Ad-lacZ immunization (Bolinger et al., 2013; GEO: GSE73314).

Combined Analysis Reveals a Conserved Expression Profile and Phenotype

Gene set enrichment analysis (GSEA) confirmed enrichment of the significantly up- and downregulated genes of the inflating βgal_{96}- and M38-specific $CD8^+$ T cells (Figure S2A). Overall, these comparisons revealed that M38- and βgal_{96}-specific inflating $CD8^+$ T cells shared 663 upregulated and 290 downregulated genes (compared to naive $CD8^+$ T cells; Figure S2B). We analyzed the transcriptional changes in the MCMV-induced and vaccine-induced memory pools, focusing on the cell cycle, apoptosis, TFs, and receptors for cytokines and chemokines, similar to an analysis of human CMV infection (Hertoghs et al., 2010).

For cell-cycle-related genes, the highly upregulated genes in the expanded populations in both models included MKI67 (Ki-67), a marker of proliferation. Its expression was confirmed by FACS, and both models showed a similar profile. By day 100, the levels of proliferation in the expanded population had dropped to a similar level to those in the contracted memory (Figures 3A and 3B).

For cell survival, whereas expanded T cells maintained expression of genes seen in acute infection, contracted memory exhibited a similar expression profile to that of naive $CD8^+$ T cells (Figure 3C). For example, FACS analysis of the anti-apoptotic protein Bcl-2 demonstrated downregulation in the acute phase in all responses but recovered at later time points only in the classical memory pools (Figure 3D).

"Tuning" of function through inhibitory and activation receptors occurs on T cells. We analyzed CD27 and CD357 (Watts, 2005) and confirmed reduced expression of these stimulatory receptors on expanded $CD8^+$ T cells (data not shown). Similarly, for both models, we extended previous data (Bolinger et al., 2013; Snyder et al., 2008) and showed overexpression of *Klrc1* (NKG2A), *Klrg1* (KLRG1), *Klre1*, *Klra1* (Ly-49c), and *Klrk1* (Nkg2d) in the expanded responses and unique expression of *Ly49C* mRNA, confirmed by flow cytometry analysis or qPCR (data not shown). Further analysis of inhibitory molecules associated with exhaustion (Blackburn et al., 2009; Wherry et al., 2007) revealed that the genes for PD-1, TIM3, CD160, LAG3, and BTLA were expressed after infection and immunization (data not shown). However, FACS analysis of PD1, TIM3, and BTLA showed high expression of these markers on days 7/21 followed by a gradual decrease in the memory phase to levels similar to naive $CD8^+$ T cells (Figure 3E). Consistent with retained functionality, the expression of granzymes, such as *Gzma*, *Gzmb*, *Gzmm*, and *Gzmk* were upregulated in both models, compared to naive $CD8^+$ T cells (data not shown).

Long-term maintenance of cell function is linked to cytokine signaling ("signal 3"). IL-7Rα (CD127) and IL-15Rβ (CD122) have been shown to be downregulated on inflationary $CD8^+$ T cells (Bolinger et al., 2013; Snyder et al., 2008). We confirmed downregulation of IL-7Rα as well as downregulation of IL-6Rα (Figure 4A). These data were confirmed by FACS (Figure 4B). Inflationary $CD8^+$ T cells also showed marked alterations in chemokine receptors (Figure 4A). We confirmed upregulation

of CX3CR1 and relative downregulation of CXCR3 on tetramer$^+$ CD8$^+$ T cells by FACS (Figure 4B).

Next, we further focused on the differential expression of mRNAs of TFs (Figure 4C). T-bet and Eomes, critical for T cell memory formation, were upregulated on both specificities of CD8$^+$ T cells in the acute phase: on M38- and βgal_{96}-specific CD8$^+$ T cells, Eomes then decreased to levels similar to that of naive CD8$^+$ T cells (Figure 4D). T-bet expression, however, showed divergence in the memory phase, being low on contracting CD8$^+$ T cells and remaining high on expanded CD8$^+$ T cells; after adenovector vaccination, Tbet remained high on 60% of the expanded cell population at day 200 versus <10% on contracted memory at that time point (Figure S3A). Relative expression of other TFs such as *Rog* (repressor of GATA), Blimp-1 (*Prdm1*), Bcl-6, *Id2*, and *Id3* were also consistent with this (Figure S3B; data not shown).

To define distinctions between memory populations further—and the role of TFs in driving these—we used principal-component analysis (PCA) (Figure S4). These data demonstrated that all antigen-experienced cell types cluster separately from naive cells and, compared to the "acute" sample, the classic memory pools (M45/βgal_{497}) regress toward the naive cell position whereas the expanded (M38/βgal_{96}) populations further extended the distance. The PCA analysis was confirmed when only using significantly modulated TFs (post-ANOVA test; Figure S4), indicating that the principal components separating these memory populations derived from changes in a limited number of TFs.

Finally, many of these features of MCMV-induced and adenovector-induced memory cells closely resembled data from a study of HCMV infection (Hertoghs et al., 2010). Similar GSEA profiles were seen using expression sets based on MCMV infection (confirming a recent analysis; Quinn et al., 2015) and, strikingly, adenovirus vaccination (Figure S4). Additionally, from GSEA analyses on the TF subset, we confirmed that TFs upregulated in HCMV (Hertoghs et al., 2010) are enriched in upregulated TFs post-MCMV or -adenovector, notably Tbet (ranked 1; Figure S4).

Preserved Phenotype, Function, and TF Expression in Human-Adenoviral-Vaccine-Induced Responses

To assess in depth whether such changes in T cell phenotype and in TF expression could be observed in human-adenovector-vaccine-induced responses, we analyzed further CD8$^+$ T cells primed using a chimpanzee adenovirus strategy (ChAd3), specific for dominant HCV epitopes from NS3 ($NS3_{95}$, HLA-A2 restricted and $NS3_{103}$ HLA-A1 restricted). We compared them to CMV-specific responses from the same individuals. We addressed (1) whether a CMV-like effector memory phenotype could be observed and (2) whether this was associated with a Tbet-high sustained memory subset.

First, using a CYToF data set (Swadling et al., 2014), we analyzed the distribution of effector-memory-associated phenotypic markers on CMV- and HCV-specific (i.e. vaccine-induced) cells in parallel. This demonstrated a shared phenotype and one distinct from T cell responses to the non-persistent pathogen influenza (Figure 5A), most clearly seen in analysis of CD57. Overlapping populations of CD57$^+$ CD127^{low} CD8$^+$ T cell pools can be seen in both CMV-specific and adenovirus-induced responses. PCA revealed overlapping distribution of CMV-specific and vaccine-induced CD8$^+$ T cell responses and distinct from those induced by influenza infection (Figure 5B).

Next, we analyzed the expression of two TFs—Tbet and Eomes. In bulk CD8$^+$ T cell populations, these showed associations with distinct memory pools, with highest Tbet levels in Tem and Temra pools (Figure 5C). In antigen-specific cells, we identified similar patterns of Tbet and Eomes regulation in CMV- and vaccine-induced cells, with enrichment for Tbet$^+$ Eomes$^-$ cells in the HCV-specific vaccine populations (Figure 5D). This distribution was maintained when the virus-specific cells were analyzed following subdivision using CCR7/CD45RA expression (Figure S5A) or within a single individual (Figure S5B) or comparing populations that had received only adenoviral vaccination and those where there had been boosting using an MVA construct (Figure S5C).

The data confirmed that the adenovirus-vaccine-induced responses in humans are dominated by effector memory populations. These are high in Tbet and relatively low in Eomes, similar to CMV-specific responses from the same individuals.

DISCUSSION

Memory T cells are considered long-lived cells that require little if any antigen re-encounter for survival. However, continued low-level stimulation through persistence of antigen is common in vivo. We hypothesized the latter could underlie the ability of adenoviral vectors to promote T cell responses in humans. Here, by profiling CMV-specific and adenovirus-vaccine-specific memory CD8$^+$ T cells, we identified patterns of CD8$^+$ T cell evolution that are (1) conserved between infection and vaccination and (2) consistent between mouse and man.

Memory inflation has been shown to be independent of initial immunodominance in MCMV (Karrer et al., 2004), and inflationary epitopes are immunoproteasome independent—suggesting they are not presented on DCs (Hutchinson et al., 2011). The current model is that repetitive antigen presentation occurs on a non-classical antigen-presenting cell (APC), potentially in the vascular endothelium or a non-hematopoietic (nh) lymph node cell (Smith et al., 2014; Torti et al., 2011). A separate study with a recombinant adenovirus vector concluded that nhAPC might play a role in memory (Bassett et al., 2011).

The expanded CD8$^+$ T cell pools downregulated the anti-apoptotic marker Bcl-2 compared to conventional memory CD8$^+$ T cells. However, the anti-apoptotic Bcl-X_L was uniquely upregulated. Bag3, another anti-apoptotic molecule, was highly expressed, and Bim, a pro-apoptotic marker, was downregulated (Figure 3C). Bcl-X_L expression can be induced by CX3CR1 (Boehme et al., 2000), which was highly upregulated in inflation (Figure 4B). Co-stimulation via 4-1BB has been shown to be critical for memory inflation (Humphreys et al., 2010); because 4-1BB also induces Bcl-X_L (Stärck et al., 2005), we hypothesize that both CX3CR1 and 4-1BB contribute to survival of CD8$^+$ T cells.

CD8$^+$ effector memory pools express high levels of T-bet and to a lower extent Eomes. T-bet expression is induced by TCR signaling and amplified by IL-12- and mTOR-mediated signals (Intlekofer et al., 2008). This program seems to be present in HCV-specific CD8$^+$ T cells induced using ChAd3 in volunteers. Such pools show sustained effector-memory phenotype,

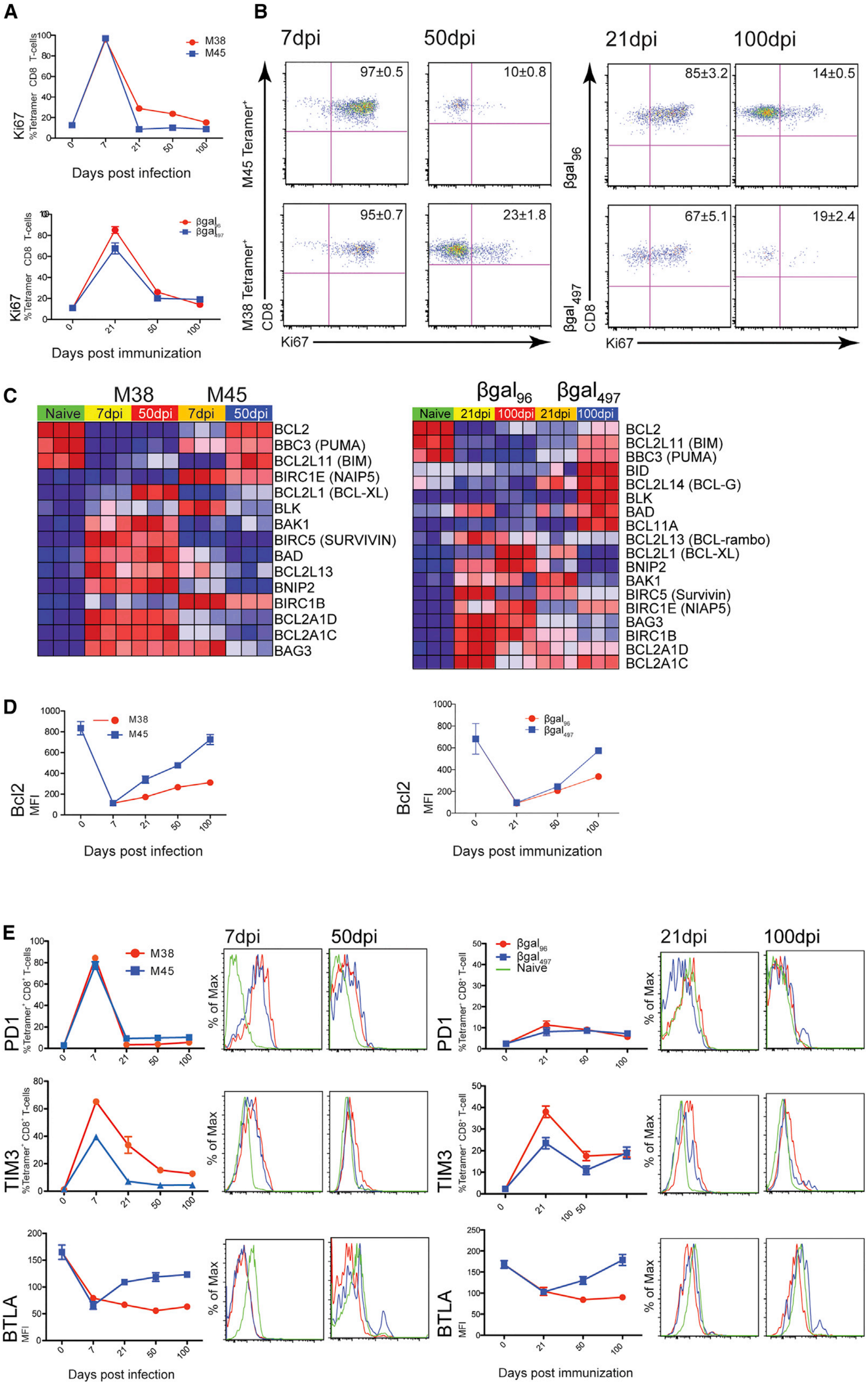

(legend on next page)

analogous to hCMV-specific responses, and a T-bet^{hi}/Eomes^{lo} status, consistent with the mouse data. This is particularly resonant for HCV-specific responses, because this is the transcriptional pattern linked with clearance of virus (Paley et al., 2012). The frequencies of responses induced by vaccination in humans do not reach those seen in "inflationary" models; however, they do reflect frequencies seen with intramuscular as opposed to (optimized) i.v. inoculation, with the same phenotypic/transcriptional changes noted (Bolinger et al., 2013).

Overall, we have identified gene sets modulated in CD8$^+$ T cells post-adenoviral vaccine compared to acute effector CD8$^+$ T cells and to conventional memory CD8$^+$ T cells. By comparing these to the "inflating" CD8$^+$ T cells from MCMV-infected animals, we show the CD8$^+$ T cell gene profile is distinct from that seen in exhaustion and conventional memory and sustains a memory development program likely linked with low-level antigen persistence. Such CD8$^+$ T cells possess a conserved phenotypic and functional pattern, underpinned by a core set of TFs. We show that this program of memory induction is reproduced in chimpanzee-adenovirus clinical vaccine trials, with implications for immunity induced by such regimens in humans.

EXPERIMENTAL PROCEDURES

Ethics Statement

Mouse experiments were performed according to UK Home Office regulations (project license number PPL 30/2235 and 30/2744) and after review and approval by the local ethical review board at the University of Oxford.

Viruses

MCMV strain (Strain Smith; ATCC: VR194) was used and kindly provided by Professor U.H. Koszinoswki, Department of Virology, Max von Pettenkofer Institute. MCMV was propagated and titrated on NIH 3T3 cells (ECACC), stored at −80°C, and injected i.v. at a dose of 2 × 10^6 pfus.

Adenoviral Vector

Recombinant adenovirus expressed the βgal protein under the control of the human CMV promoter (Ad-LacZ; Krebs et al., 2005). Ad-LacZ was propagated on HER-911 cells and purified with the Vivapure AdenoPack 20 (Sartorius; Stedim Biotech). Virus titer was determined in a cytopathic effect assay (Krebs et al., 2005). Ad-LacZ was stored at −80°C in PBS and injected i.v. at a dose of 2 × 10^9 pfus.

Mice

C57BL/6 mice were obtained from Harlan, kept under conventional conditions in individually ventilated cages, and fed with normal chow diet.

Peptides

Peptides were MCMV, M38$_{316–323}$ (SSPPMFRV; Munks et al., 2006), and M45$_{985–993}$ (HGIRNASFI; Gold et al., 2002; Proimmune) and βgal$_{96–103}$ (DAPIYTNV; Overwijk et al., 1997) and βgal$_{497–504}$ (ICPMYARV; Oukka et al., 1996; Mimotopes).

Antibodies

Antibodies were obtained from eBioscience, BD Bioscience, BioLegend, Abcam, R&D Systems, and Jackson ImmunoResearch Laboratories.

Flow Cytometry

Single-cell suspensions were generated from the indicated organs, and 1 × 10^6 cells were incubated with the indicated mAb at 4°C for 20 min. For PBL samples, erythrocytes were lysed with FACS Lysing Solution (BD PharMingen). Cells were analyzed by flow cytometry using a BD LSR II flow cytometer and FlowJo (Treestar), gated on viable leukocytes using the live/dead fixable near-IR dead cell stain kit from Invitrogen.

Intracellular Staining and Peptide Stimulation

Following surface staining, cells were fixed and permeabilized using the FOXP3 Fixation/Permeabilisation Kit from eBioscience. Cells were resuspended in permeabilization buffer containing the appropriate amount of antibody and incubated for 30 min at 4°C. For peptide stimulation, see Supplemental Experimental Procedures.

Construction of Tetrameric MHC Class I Peptide Complexes

MHC class I monomers complexed with M38 (H-2Kb), M45 (H-2Db), and βgal (H-2Kb) were produced as previously (Altman et al., 1996) and tetramerized by addition of streptavidin-PE (BD Bioscience) or streptavidin-APC (Invitrogen). Aliquots of 1 × 10^6 cells or 100 μl of whole blood were stained using 50 μl of a solution containing tetrameric class I peptide complexes at 37°C for 20 min followed by staining with mAbs.

Sorting Cells by MACS

CD8$^+$ T cells were isolated from spleens and single-cell suspensions prepared, resuspended in MACS buffer at a concentration of 10^7 cells/90 μl, and purified by negative selection using the CD8$^+$ T cell selection kit/AutoMACS (Miltenyi Biotec).

Cell Sorting by MoFLO

For cell sorting, at least four spleens were pooled per sort using MoFlo (Beckman Coulter Genomics). Splenocyte samples depleted of erythrocytes and enriched for CD8$^+$ T cells using MACS beads were stained with saturating concentrations of antibody and tetramer, incubated for 1 hr on a rolling platform at 4°C, washed, filtered, and resuspended. Sorted cells were pelleted at 9,000 rpm for 10 min and snap frozen using methanol and dry ice. Reanalysis of sorted cells indicated a purity of over 95%.

Figure 3. Turnover, Survival, and Expression of Inhibitory Receptors on Sustained Memory CD8$^+$ T Cell Pools

C57BL/6 mice were infected i.v. with 1 × 10^6 pfus MCMV or immunized i.v. with 1 × 10^9 pfus Ad-LacZ.

(A) Longitudinal analysis of the percentage of cells expressing Ki67 on MCMV-specific (left panel) and βgal-specific CD8$^+$ T cells (right panel) in the spleen on days 0, 7, 21, 50, and 100 postinfection or postvaccination, measured by flow cytometry (n = 6–8; ±SEM).

(B) Representative flow cytometry plots show the expression of Ki67 in the spleen on days 7 and 50 postinfection or d21 and d100 postvaccination gated on live M38- or M45-specific (left panel) and βgal$_{96}$- or βgal$_{497}$-specific (right panel) CD8 T cells.

(C) Heatmap showing mRNA expression levels of proteins involved in apoptosis measured by microarray analysis of naive, M38-, and M45-specific CD8$^+$ T cells days 7 and 50 postinfection (left panel) and naive CD8$^+$ T cells, βgal$_{96}$-, and βgal$_{497}$-specific CD8$^+$ T cells days 21 and 100 postvaccination (right panel). Shades of red indicate upregulated genes, and shades of blue indicate downregulated genes. Filter criteria of at least 2-fold changes with $p \leq 0.05$, compared to naive CD8$^+$ T cells, are shown.

(D) Longitudinal analysis of the percentage of cells expressing Bcl2 on MCMV-specific (left panel) and βgal-specific CD8$^+$ T cells (right panel) in the spleen on days 0, 7, 21, 50, and 100 postinfection or postvaccination, measured by flow cytometry (n = 6–8; ±SEM).

(E) Flow cytometry analysis showing the percentage or the MFI of cells expressing the inhibitory receptors PD1, TIM3, and BTLA on M38- (red) and M45-specific CD8$^+$ T cells (blue; n = 6–8; ±SEM) and on βgal$_{96}$- (red) and βgal$_{497}$-specific (blue) CD8$^+$ T cells in the spleen (n = 6–8; ±SEM). Histograms show expression of PD-1, TIM-3, and BTLA and are gated on live naive (green) M38/βgal$_{96}$-specific (red) and M45/βgal$_{497}$-specific CD8$^+$ T cells (blue).

OPEN
ACCESS
CellPress

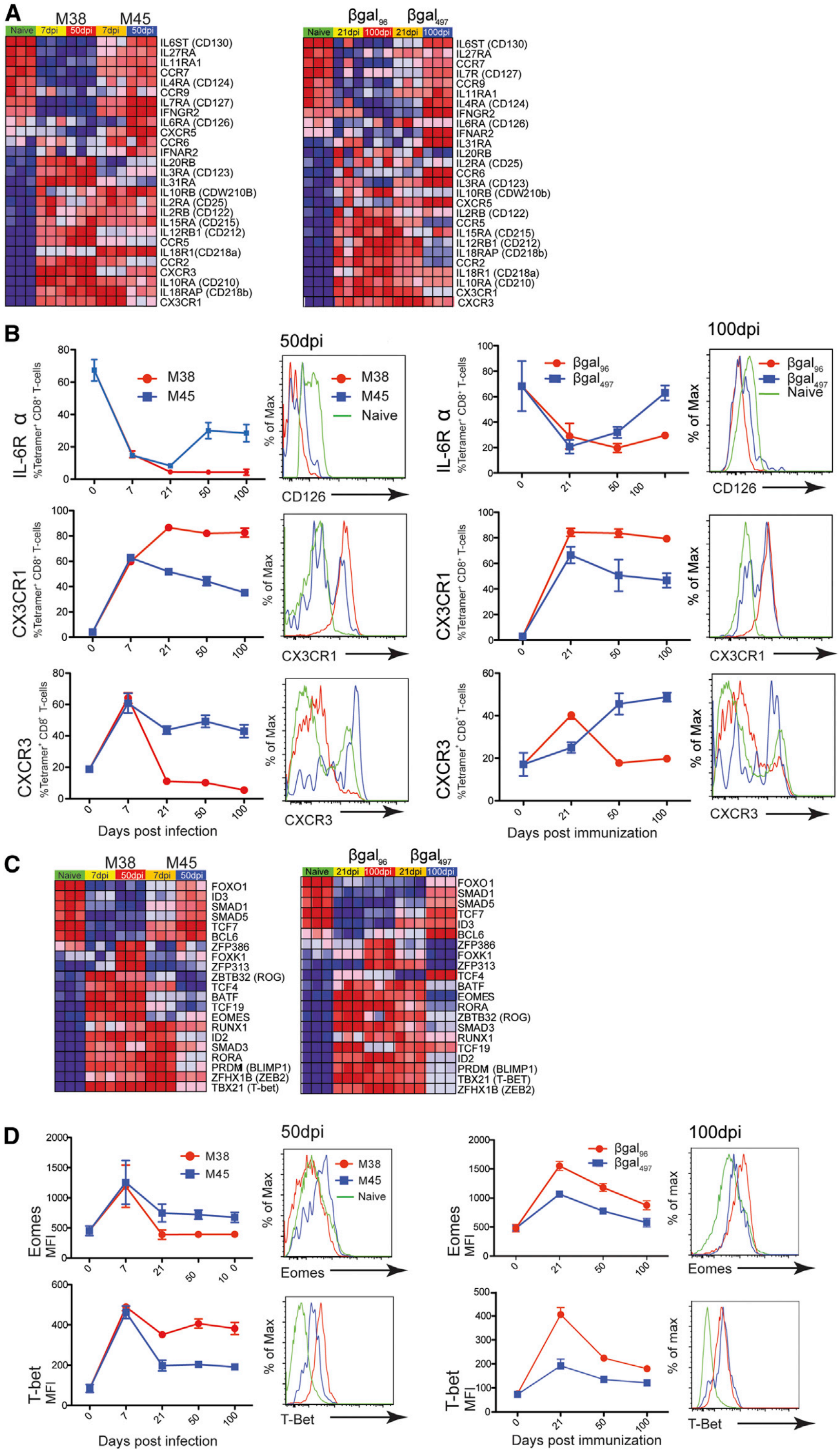

Figure 4. Distinct Cytokine and Chemokine Receptor Expression and Individual Transcription Regulator Profile on Expanded CD8$^+$ T Cells Compared to Conventional Memory CD8$^+$ T Cells

(A) Heatmap showing mRNA expression levels of cytokine and chemokine receptors measured by microarray analysis of naive, M38-, and M45-specific CD8$^+$ T cells days 7 and 50 postinfection (left panel) and naive, βgal_{96}-, and βgal_{497}-specific CD8$^+$ T cells days 21 and 100 postvaccination (right panel). Shades of red indicate upregulated genes, and shades of blue indicate downregulated genes. Filter criteria of at least 2-fold changes with $p \leq 0.05$, compared to naive CD8$^+$ T cells, are shown.

(B) Longitudinal flow cytometry analysis showing the percentage of IL-6Rα (CD126), CX3CR1, and CXCR3 expression on M38/βgal_{96}- (red) and M45/βgal_{497}-specific CD8$^+$ T cells (blue) in the spleen at days 0, 7, 21, 50, and 100 postinfection/immunization (n = 6–8; ±SEM). Histograms show expression of indicated markers, gated on live naive (green) M38/βgal_{96}- (red) and M45/βgal_{497}-specific CD8$^+$ T cells (blue).

(C) Heatmap showing mRNA expression levels of transcriptional regulators measured by microarray analysis of naive, M38-, and M45-specific CD8$^+$ T cells days 7 and 50 postinfection (left panel) and naive CD8$^+$ T cells and βgal_{96}- and βgal_{497}-specific CD8$^+$ T cells days 21 and 100 postvaccination (right panel). Shades of red indicate upregulated genes, and shades of blue indicate downregulated genes. Filter criteria of at least 2-fold changes with $p \leq 0.05$, compared to naive CD8$^+$ T cells, are shown.

(D) Flow cytometry analysis showing the MFI of cells expressing the TFs EOMES and Tbet on M38- (red) and M45-specific CD8$^+$ T cells (blue; n = 6–8; ±SEM) and on βgal_{96}- (red) and βgal_{497}-specific (blue) CD8$^+$ T cells in the spleen (n = 5–9; ±SEM). Histograms show expression of EOMES and Tbet and are gated on live naive (green), M38/βgal_{96}-specific CD8$^+$ T cells (red), and M45/βgal_{497}-specific CD8$^+$ T cells (blue).

See also Figures S3 and S4.

RNA Extraction and Microarray Analyses

Total RNA was isolated from sorted cells using the mirVana Kit (Ambion). RNA integrity was checked using Agilent 2100 Bioanalyzer, and samples with a RIN value of 8 or more were used for amplification. cDNA was synthesized and cRNA made using Illumina Total Prep RNA amplification Kit (Ambion). Samples were hybridized to Illumina MouseWG-6 v2.0. For microarray data analysis, see the Supplemental Experimental Procedures.

Statistical Analysis of Real-Time and Flow Cytometry Data

Unpaired two-tailed Student's test was used. Statistical data analysis was performed using Graph-Pad Prism version 5.0a for MACs (GraphPad Software).

Human Vaccination Protocols

The Ad6, ChAd3, and MVA vectors encoding the NS3-5B region of genotype 1B (Ad6-NSmut, based on sequence accession number M58335) and vaccination schedules have been described previously (Barnes et al., 2012; Swadling et al., 2014; ClinicalTrial.gov database ID: NCT01070407 and NCT01296451).

Human Pentamer Staining and Flow Cytometry

Pentamer staining was as previously (Swadling et al., 2014). For staining details, see the Supplemental Experimental Procedures.

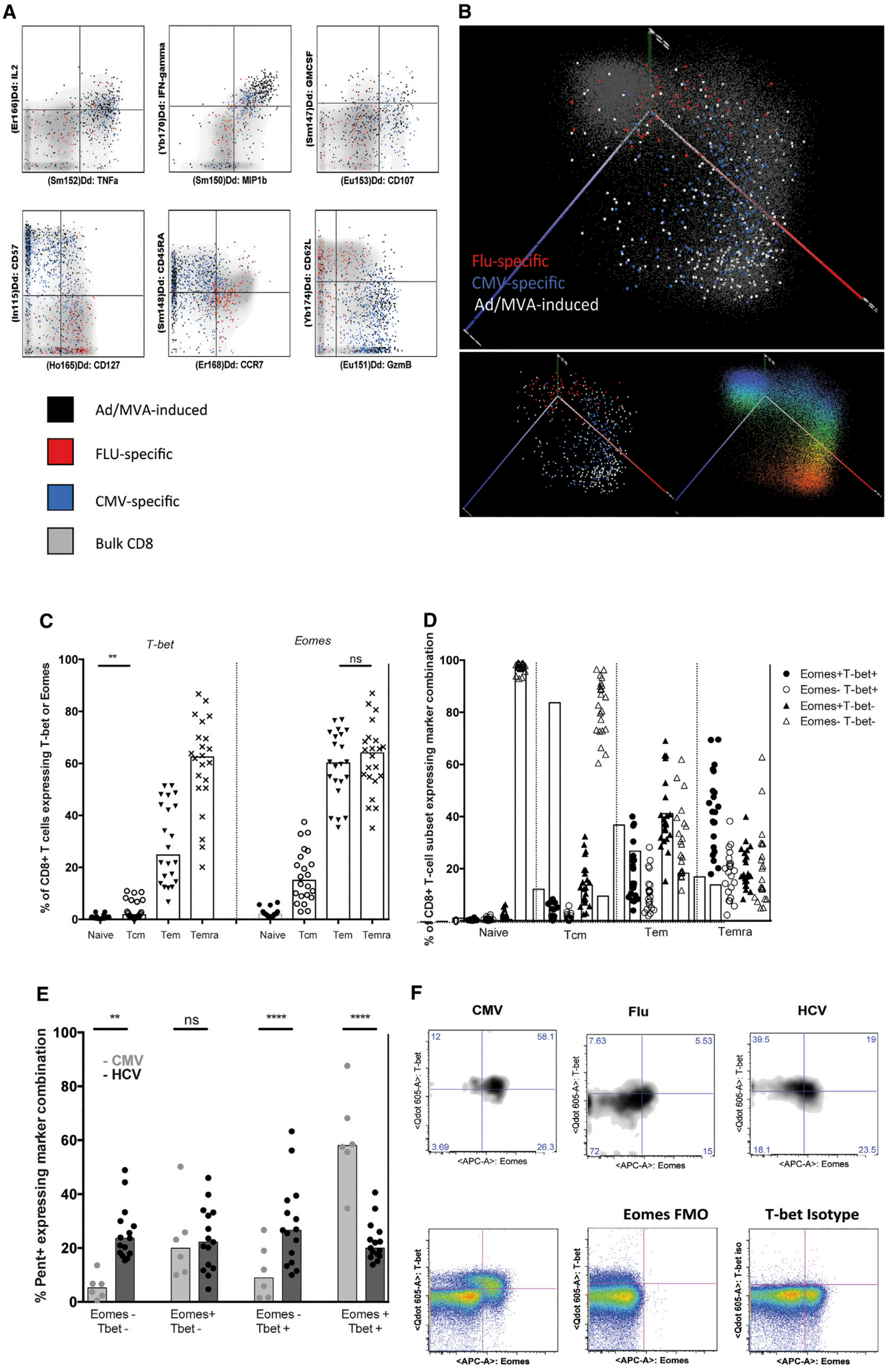

(legend on next page)

Mass Cytometry Using CyTOF

Detailed methodology and validation of the mass cytometry used here are described elsewhere (Newell et al., 2012). T cell phenotype and function were determined by mass cytometry analysis on frozen PBMCs from two volunteers receiving ChAd3-NSmut and MVA-NSmut. Samples were taken 14 weeks after MVA-NSmut boost vaccination. Two healthy controls each for CMV and FLU, previously shown to have a detectable T cell response by pentamer FACS staining, were also included. For PCA details, see Supplemental Experimental Procedures.

SUPPLEMENTAL INFORMATION

Supplemental Information includes Supplemental Experimental Procedures and five figures and can be found with this article online at http://dx.doi.org/10.1016/j.celrep.2015.10.034.

AUTHOR CONTRIBUTIONS

S.S., B.B., L.S., G.O., C.d.L., L.N.L., and D.B. performed the mouse functional and microarray experiments; N.S. and E.M. contributed to the bioinformatics analyses; and M.D., E.N., L.S., S.C., A.F., and E.B. contributed to the human vaccines studies. S.S., P.K., and B.B. analyzed the data and finalized the manuscript. S.S. and B.B. share co-authorship for contributions to design, execution, and interpretation of the murine MCMV and adenovirus experiments.

ACKNOWLEDGMENTS

The project received support from the Oxford Martin School, the Wellcome Trust (WT091663MA), the Swiss National Science Foundation, the *Schweizerische Stiftung für Medizinisch-Biologische Stipendien* (SSMBS), NIH U19AI082630, the MRC (SF to E.B.), and the NIHR Biomedical Research Centre, Oxford. We thank Alasdair J. Lesley for running the MoFlo and the Wellcome Trust Centre for Human Genetics.

Received: June 2, 2014
Revised: September 9, 2015
Accepted: October 10, 2015
Published: November 12, 2015

REFERENCES

Altman, J.D., Moss, P.A.H., Goulder, P.J.R., Barouch, D.H., McHeyzer-Williams, M.G., Bell, J.I., McMichael, A.J., and Davis, M.M. (1996). Phenotypic analysis of antigen-specific T lymphocytes. Science *274*, 94–96.

Barnes, E., Folgori, A., Capone, S., Swadling, L., Aston, S., Kurioka, A., Meyer, J., Huddart, R., Smith, K., Townsend, R., et al. (2012). Novel adenovirus-based vaccines induce broad and sustained T cell responses to HCV in man. Sci. Transl. Med. *4*, 115ra1.

Bassett, J.D., Yang, T.C., Bernard, D., Millar, J.B., Swift, S.L., McGray, A.J., VanSeggelen, H., Boudreau, J.E., Finn, J.D., Parsons, R., et al. (2011). CD8+ T-cell expansion and maintenance after recombinant adenovirus immunization rely upon cooperation between hematopoietic and nonhematopoietic antigen-presenting cells. Blood *117*, 1146–1155.

Blackburn, S.D., Shin, H., Haining, W.N., Zou, T., Workman, C.J., Polley, A., Betts, M.R., Freeman, G.J., Vignali, D.A., and Wherry, E.J. (2009). Coregulation of CD8+ T cell exhaustion by multiple inhibitory receptors during chronic viral infection. Nat. Immunol. *10*, 29–37.

Boehme, S.A., Lio, F.M., Maciejewski-Lenoir, D., Bacon, K.B., and Conlon, P.J. (2000). The chemokine fractalkine inhibits Fas-mediated cell death of brain microglia. J. Immunol. *165*, 397–403.

Bolinger, B., Sims, S., O'Hara, G., de Lara, C., Tchilian, E., Firner, S., Engeler, D., Ludewig, B., and Klenerman, P. (2013). A new model for CD8+ T cell memory inflation based upon a recombinant adenoviral vector. J. Immunol. *190*, 4162–4174.

Colloca, S., Barnes, E., Folgori, A., Ammendola, V., Capone, S., Cirillo, A., Siani, L., Naddeo, M., Grazioli, F., Esposito, M.L., et al. (2012). Vaccine vectors derived from a large collection of simian adenoviruses induce potent cellular immunity across multiple species. Sci. Transl. Med. *4*, 115ra2.

Gold, M.C., Munks, M.W., Wagner, M., Koszinowski, U.H., Hill, A.B., and Fling, S.P. (2002). The murine cytomegalovirus immunomodulatory gene m152 prevents recognition of infected cells by M45-specific CTL but does not alter the immunodominance of the M45-specific CD8 T cell response in vivo. J. Immunol. *169*, 359–365.

Hansen, S.G., Ford, J.C., Lewis, M.S., Ventura, A.B., Hughes, C.M., Coyne-Johnson, L., Whizin, N., Oswald, K., Shoemaker, R., Swanson, T., et al. (2011). Profound early control of highly pathogenic SIV by an effector memory T-cell vaccine. Nature *473*, 523–527.

Hertoghs, K.M., Moerland, P.D., van Stijn, A., Remmerswaal, E.B., Yong, S.L., van de Berg, P.J., van Ham, S.M., Baas, F., ten Berge, I.J., and van Lier, R.A. (2010). Molecular profiling of cytomegalovirus-induced human CD8+ T cell differentiation. J. Clin. Invest. *120*, 4077–4090.

Humphreys, I.R., Lee, S.W., Jones, M., Loewendorf, A., Gostick, E., Price, D.A., Benedict, C.A., Ware, C.F., and Croft, M. (2010). Biphasic role of 4-1BB in the regulation of mouse cytomegalovirus-specific CD8(+) T cells. Eur. J. Immunol. *40*, 2762–2768.

Figure 5. Mass Cytometry and FACS Reveal a Shared Phenotype and TF Expression of Human T Cells Induced by Virally Vectored Vaccines and CMV

(A and B) The memory T cell response induced by heterologous prime-boost vaccination with ChAd3-NSmut and MVA-NSmut were compared with that seen after natural infection with flu and CMV by CyTOF. (A) Example plots of intracellular cytokine staining of stimulated or surface and internal markers on unstimulated cells are shown. Bulk CD8s are shown as density plots in gray, and Ag-specific cells (n = 2) are overlaid as black (14 weeks post-MVA boost vaccination), red (flu-specific), or blue dots (CMV-specific). (B) 3D-PCA of vaccine-induced $CD8^+$ T cells is shown. The first three principal components were plotted in PyMOL (PC1 axis in red, PC2 axis in green, and PC3 axis in blue). In the top figure, gray dots represent single bulk CD8 T cells. Ag-specific cells (n = 2) are overlaid as white (14 weeks post-MVA boost vaccination), red (flu-specific), or blue (CMV-specific) dots. Ag-specific cells alone are shown in the bottom left panel. In the bottom right panel, the bulk $CD8^+$ T cells are shown, colored according to their relative expression of the marker CD57 in $CD8^+$ T cells, from the highest expression in red to the lowest in blue.

(C and D) Eomes and T-bet expression on bulk $CD8^+$ T cells by FACS (C) and their co-expression on $CD8^+$ T cell subsets (naive: $CD45RA^+CCR7^+$; Tcm: $CD45RA^-CCR7^+$; Tem: $CD45RA^-CCR7^-$; Temra: $CD45RA^+CCR7^-$; n = 22; D). Bars are at median. All comparisons of T-bet and Eomes expression between any two T cell subsets were highly significant ($p < 0.0001$****) unless stated.

(E) HCV-specific memory $CD8^+$ T cells induced by virally vectored vaccine regimes (black; 8–26 weeks post-boost vaccination) or by natural infection with CMV (gray) were identified by pentamer staining, and their expression of T-bet and Eomes was assessed (CMV n = 8; Ad/Ad vaccination n = 8; Ad/MVA n = 6). Bars are at median.

(F) Example FACS plots showing T-bet versus Eomes co-staining and gating controls.

See also Figure S5.

Hutchinson, S., Sims, S., O'Hara, G., Silk, J., Gileadi, U., Cerundolo, V., and Klenerman, P. (2011). A dominant role for the immunoproteasome in CD8+ T cell responses to murine cytomegalovirus. PLoS ONE *6*, e14646.

Intlekofer, A.M., Banerjee, A., Takemoto, N., Gordon, S.M., Dejong, C.S., Shin, H., Hunter, C.A., Wherry, E.J., Lindsten, T., and Reiner, S.L. (2008). Anomalous type 17 response to viral infection by CD8+ T cells lacking T-bet and eomesodermin. Science *321*, 408–411.

Karrer, U., Sierro, S., Wagner, M., Oxenius, A., Hengel, H., Koszinowski, U.H., Phillips, R.E., and Klenerman, P. (2003). Memory inflation: continuous accumulation of antiviral CD8+ T cells over time. J. Immunol. *170*, 2022–2029.

Karrer, U., Wagner, M., Sierro, S., Oxenius, A., Hengel, H., Dumrese, T., Freigang, S., Koszinowski, U.H., Phillips, R.E., and Klenerman, P. (2004). Expansion of protective CD8+ T-cell responses driven by recombinant cytomegaloviruses. J. Virol. *78*, 2255–2264.

Krebs, P., Scandella, E., Odermatt, B., and Ludewig, B. (2005). Rapid functional exhaustion and deletion of CTL following immunization with recombinant adenovirus. J. Immunol. *174*, 4559–4566.

Munks, M.W., Gold, M.C., Zajac, A.L., Doom, C.M., Morello, C.S., Spector, D.H., and Hill, A.B. (2006). Genome-wide analysis reveals a highly diverse CD8 T cell response to murine cytomegalovirus. J. Immunol. *176*, 3760–3766.

Newell, E.W., Sigal, N., Bendall, S.C., Nolan, G.P., and Davis, M.M. (2012). Cytometry by time-of-flight shows combinatorial cytokine expression and virus-specific cell niches within a continuum of CD8+ T cell phenotypes. Immunity *36*, 142–152.

Oukka, M., Cohen-Tannoudji, M., Tanaka, Y., Babinet, C., and Kosmatopoulos, K. (1996). Medullary thymic epithelial cells induce tolerance to intracellular proteins. J. Immunol. *156*, 968–975.

Overwijk, W.W., Surman, D.R., Tsung, K., and Restifo, N.P. (1997). Identification of a Kb-restricted CTL epitope of beta-galactosidase: potential use in development of immunization protocols for "self" antigens. Methods *12*, 117–123.

Paley, M.A., Kroy, D.C., Odorizzi, P.M., Johnnidis, J.B., Dolfi, D.V., Barnett, B.E., Bikoff, E.K., Robertson, E.J., Lauer, G.M., Reiner, S.L., and Wherry, E.J. (2012). Progenitor and terminal subsets of CD8+ T cells cooperate to contain chronic viral infection. Science *338*, 1220–1225.

Quinn, M., Turula, H., Tandon, M., Deslouches, B., Moghbeli, T., and Snyder, C.M. (2015). Memory T cells specific for murine cytomegalovirus re-emerge after multiple challenges and recapitulate immunity in various adoptive transfer scenarios. J. Immunol. *194*, 1726–1736.

Sallusto, F., Lenig, D., Förster, R., Lipp, M., and Lanzavecchia, A. (1999). Two subsets of memory T lymphocytes with distinct homing potentials and effector functions. Nature *401*, 708–712.

Sierro, S., Rothkopf, R., and Klenerman, P. (2005). Evolution of diverse antiviral CD8+ T cell populations after murine cytomegalovirus infection. Eur. J. Immunol. *35*, 1113–1123.

Smith, C.J., Turula, H., and Snyder, C.M. (2014). Systemic hematogenous maintenance of memory inflation by MCMV infection. PLoS Pathog. *10*, e1004233.

Snyder, C.M., Cho, K.S., Bonnett, E.L., van Dommelen, S., Shellam, G.R., and Hill, A.B. (2008). Memory inflation during chronic viral infection is maintained by continuous production of short-lived, functional T cells. Immunity *29*, 650–659.

Stärck, L., Scholz, C., Dörken, B., and Daniel, P.T. (2005). Costimulation by CD137/4-1BB inhibits T cell apoptosis and induces Bcl-xL and c-FLIP(short) via phosphatidylinositol 3-kinase and AKT/protein kinase B. Eur. J. Immunol. *35*, 1257–1266.

Swadling, L., Capone, S., Antrobus, R.D., Brown, A., Richardson, R., Newell, E.W., Halliday, J., Kelly, C., Bowen, D., Fergusson, J., et al. (2014). A human vaccine strategy based on chimpanzee adenoviral and MVA vectors that primes, boosts, and sustains functional HCV-specific T cell memory. Sci. Transl. Med. *6*, 261ra153.

Torti, N., Walton, S.M., Brocker, T., Rülicke, T., and Oxenius, A. (2011). Non-hematopoietic cells in lymph nodes drive memory CD8 T cell inflation during murine cytomegalovirus infection. PLoS Pathog. *7*, e1002313.

Watts, T.H. (2005). TNF/TNFR family members in costimulation of T cell responses. Annu. Rev. Immunol. *23*, 23–68.

Wherry, E.J., Ha, S.J., Kaech, S.M., Haining, W.N., Sarkar, S., Kalia, V., Subramaniam, S., Blattman, J.N., Barber, D.L., and Ahmed, R. (2007). Molecular signature of CD8+ T cell exhaustion during chronic viral infection. Immunity *27*, 670–684.

Cell Reports

Supplemental Information

Adenoviral Vector Vaccination Induces a Conserved Program of CD8$^+$ T Cell Memory Differentiation in Mouse and Man

Beatrice Bolinger, Stuart Sims,, Leo Swadling, Geraldine O’Hara, Catherine de Lara, Dilair Baban, Natasha Saghal, Lian Ni Lee, Emanuele Marchi, Mark Davis, Evan Newell, Stefania Capone, Antonella Folgori, Ellie Barnes, and Paul Klenerman

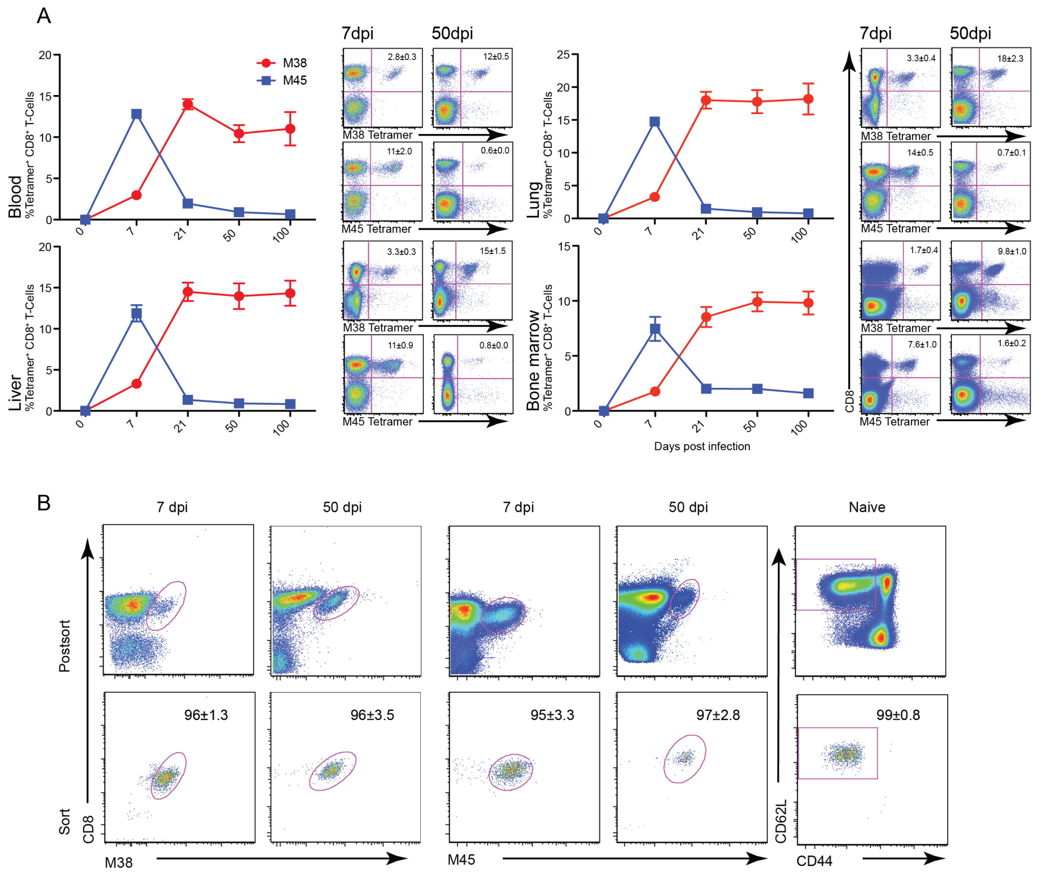

Figure S1: MCMV-specific inflating CD8 T cells are not restricted to the spleen (related to Figure 1).

C57BL/6 mice were infected i.v. with 1x10^6 pfu MCMV.

(A) Longitudinal flow cytometry analysis showing the percentage of M38- (red) and M45- (blue) specific $CD8^+$ T cells in blood, liver, lung and bone marrow at 0, 7, 21, 50 and 100 days post infection. Mean percentage of live tetramer-positive $CD8^+$ lymphocytes is indicated (n=8, mean±SEM). Representative flow cytometry plots of M38- and M45-specific $CD8^+$ T cells 7 and 50 days post infection in blood, liver, lung and bone marrow.

(B) Representative flow cytometry plots from cell sorts of M38- and M45-specific $CD8^+$ T cells 7 and 50 days post infection. Cell sorts were performed in triplicates; five mice were pooled per group. Mean percentage of live tetramer-positive $CD8^+$ lymphocytes is indicated (n=3, mean±SEM).

A

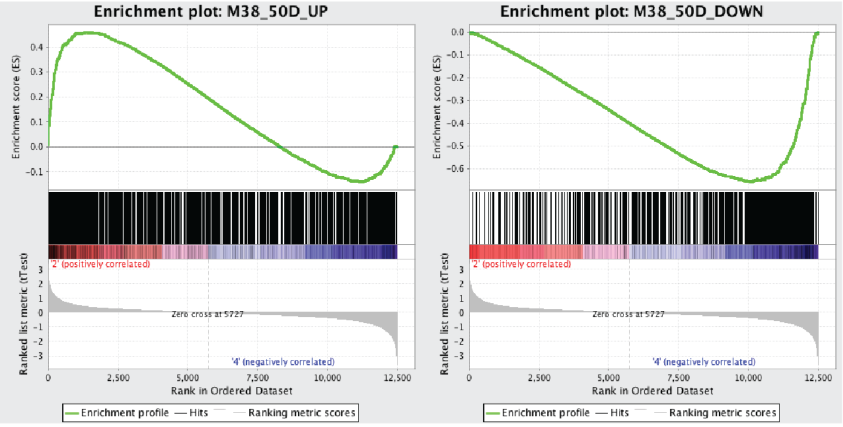

B

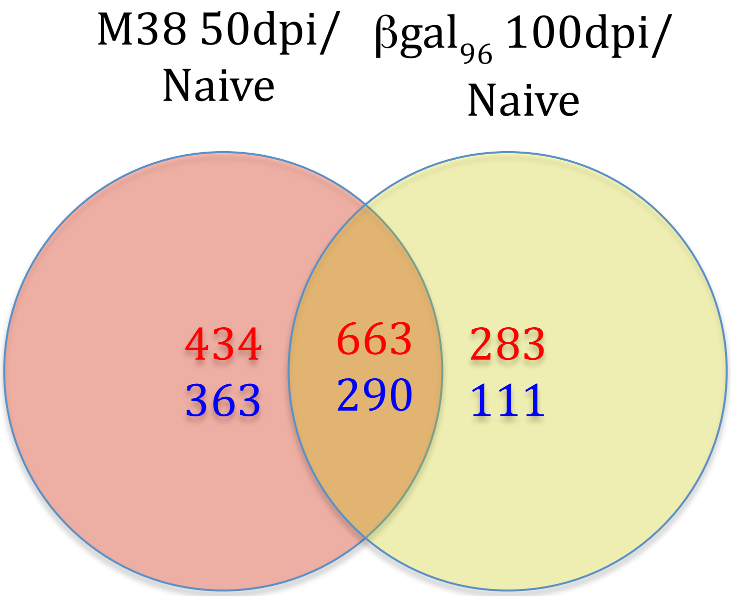

Figure S2: MCMV- and βgal-specific expanded CD8 T cells display similarities in the gene expression profile (related to Figure 2).

(A) Gene set enrichment analysis (GSEA) of genes of sustained MCMV-specific and Ad-LacZ-specific $CD8^+$ T cells. (B) Venn diagram showing the number of differentially expressed genes between βgal_{96}- specific $CD8^+$ T cells 100 (yellow) days post immunization and M38-specific $CD8^+$ T cells 50 (red) days post infection. Filter criteria of at least 2 fold change with P≤0.05

compared to naïve $CD8^+$ T cells. Up-regulated genes are indicated in red and down-regulated genes in blue.

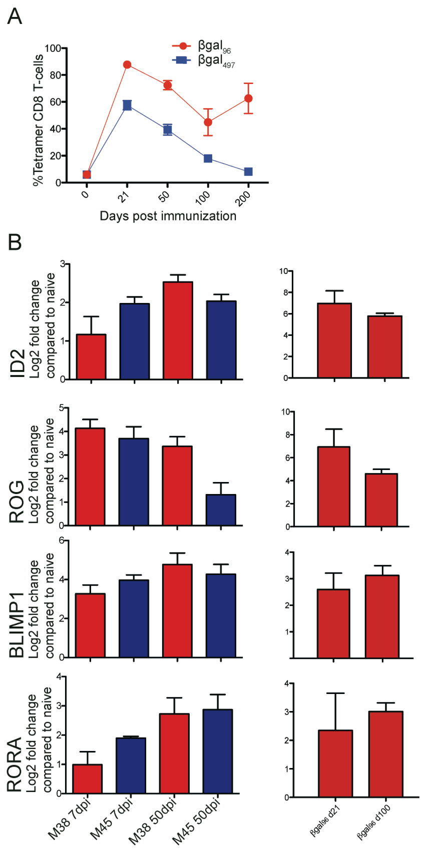

Figure S3: Tbet on βgal-specific CD8$^+$ T cell; mRNA expression of the transcriptional regulators ID2, ROG, BLIMP1 and RORA (related to Figure 4).

(A) Flow cytometry analysis showing the percentage of CD8$^+$ T cells expressing the transcription factor Tbet on βgal$_{96}$- (red) and βgal$_{497}$-specific (blue) CD8$^+$ T cells in the spleen (n=5-9, ±SEM).

(B) ID2, ROG, BLIMP1 and RORA mRNA expression was determined by quantitative RT-PCR in M38- and M45-specific CD8$^+$ T cells at day 7 and 50 post infection and in βgal$_{96}$-specific CD8$^+$ T cells d21 and d100 after immunization. Five mice were pooled for each group; real-time analysis, performed in triplicates. Data presented as Log2 fold change compared to naive CD8$^+$ T-cells after normalization against the internal control gene HPRT.

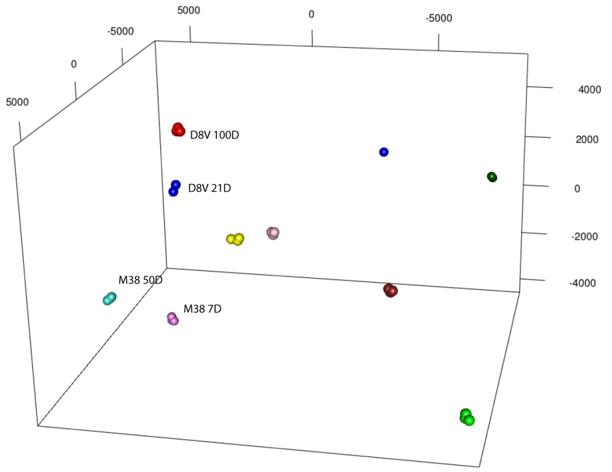

D8V 100D
D8V 21D
M38 50D
M38 7D

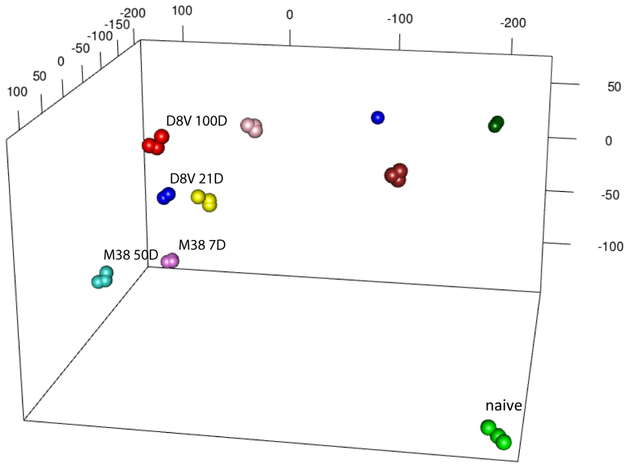

D8V 100D
D8V 21D
M38 50D
M38 7D
naive

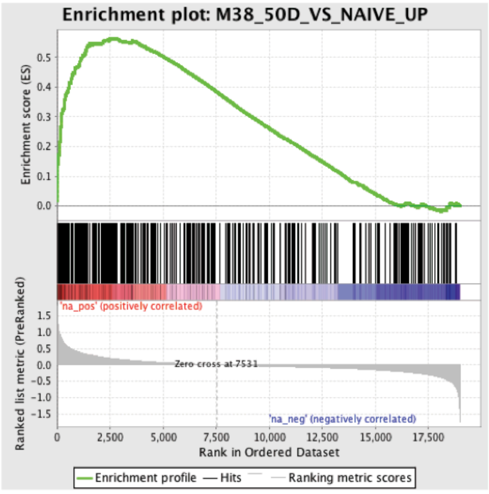

Enrichment plot: M38_50D_VS_NAIVE_UP
Enrichment score (ES)
Ranked list metric (PreRanked)
'na_pos' (positively correlated)
Zero cross at 7531
'na_neg' (negatively correlated)
Rank in Ordered Dataset
Enrichment profile — Hits — Ranking metric scores

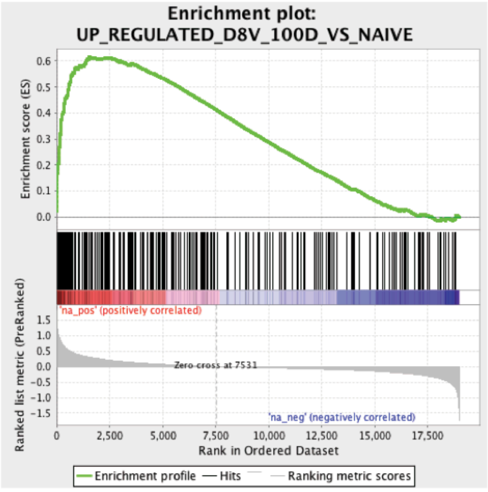

Enrichment plot:
UP_REGULATED_D8V_100D_VS_NAIVE
Enrichment score (ES)
Ranked list metric (PreRanked)
'na_pos' (positively correlated)
Zero cross at 7531
'na_neg' (negatively correlated)
Rank in Ordered Dataset
Enrichment profile — Hits — Ranking metric scores

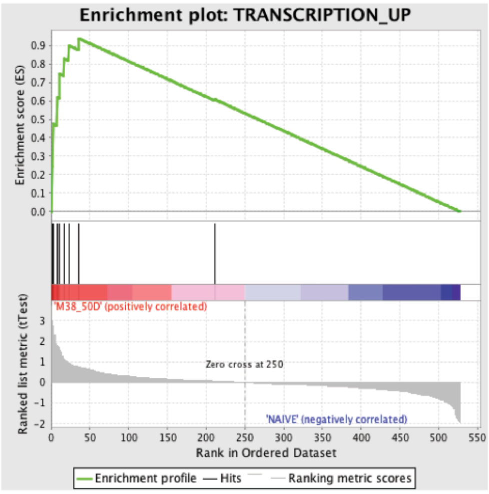

Enrichment plot: TRANSCRIPTION_UP
Enrichment score (ES)
Ranked list metric (tTest)
'M38_50D' (positively correlated)
Zero cross at 250
'NAIVE' (negatively correlated)
Rank in Ordered Dataset
Enrichment profile — Hits — Ranking metric scores

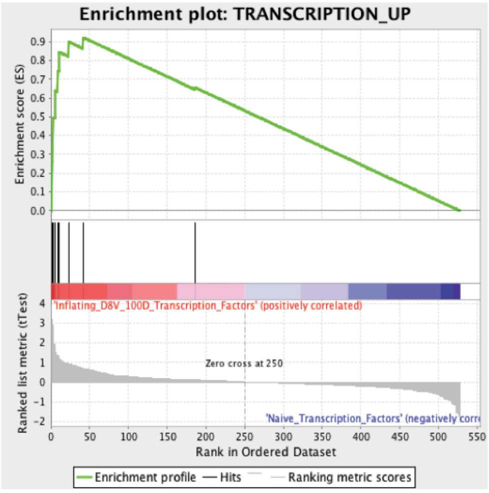

Enrichment plot: TRANSCRIPTION_UP
Enrichment score (ES)
Ranked list metric (tTest)
'Inflating_D8V_100D_Transcription_Factors' (positively correlated)
Zero cross at 250
Rank in Ordered Dataset
Enrichment profile — Hits — Ranking metric scores

Figure S4: Principal Components Analysis (PCA) and Gene set enrichment analysis (GSEA) of differential regulated genes of inflationary MCMV-specific or Ad-LacZ-specific CD8+ T cells (related to Figure 4).

Upper panels: Left hand panel shows 3D PCA of all microarray samples employing the entire set of probe intensities (following removal only of controls and weakly detected probes, 17062 total probes). The transcription profiles plotted are: naïve (green), bgal96-specific d21 (blue), bgal96-specific d100 (red), bgal497-specific d21 (yellow), bgal497-specific d100 (dark green), M38-specific d7 (violet), M38-specific d50 (turquoise), M45-specific d7 (pink) and M45-specific CD8+ T cells d50 (brown). Right hand panel is as above but PCA performed using only the subset of significantly differentially modulated genes defined as TFs (see methods).

Lower panels: GSEA was performed as indicated in the methods. The plots indicate strong, statistically robust enrichment for both up and down-regulated genes (GSEA for down-regulated genes not shown) shared between the two datasets. Left panels GSEA of genes of M38-specific and human CMV-specific inflationary CD8+ T cells, right panels GSEA of genes of bgal96-specific and human CMV-specific inflationary CD8+ T cells (Hertoghs et al., 2010). Lower panels show the same analyses using TFs only.

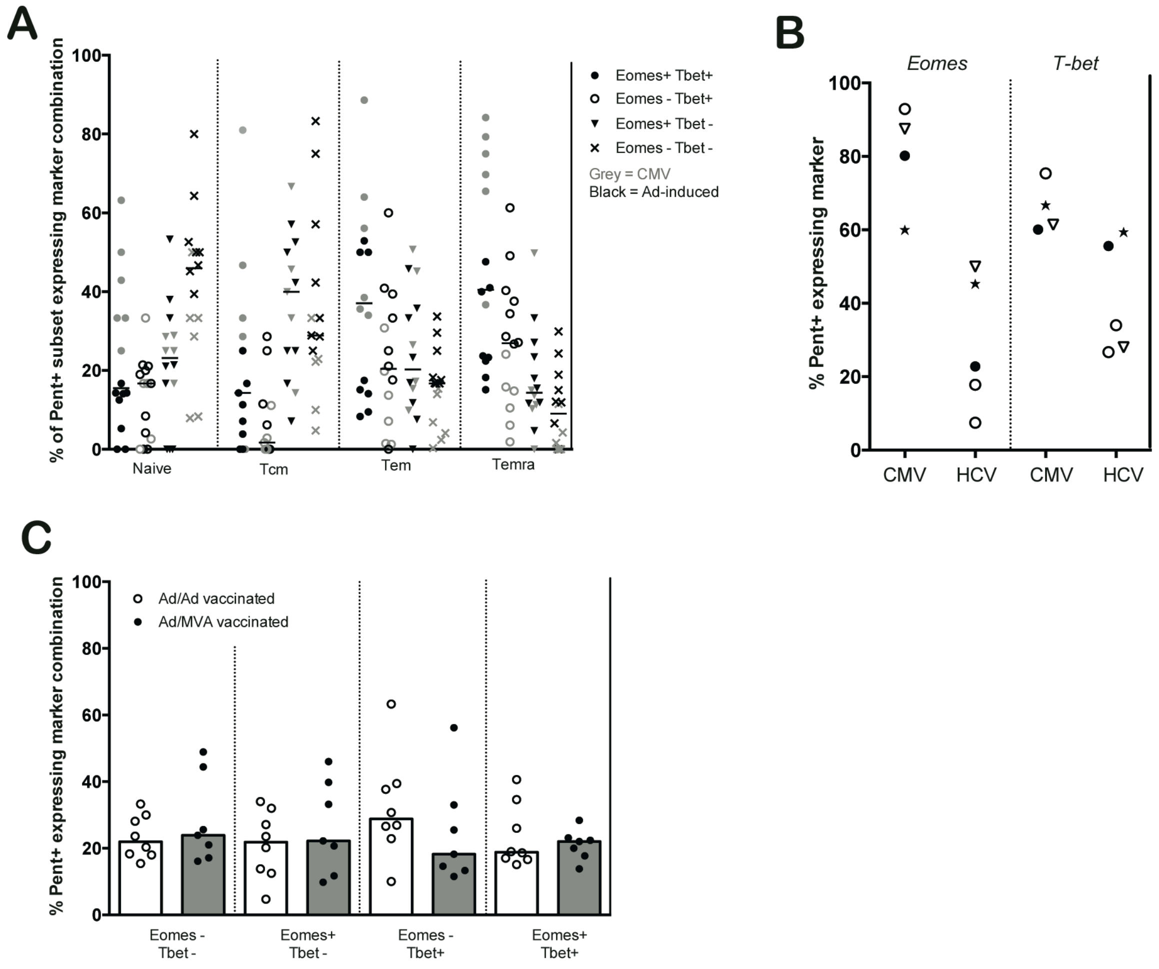

Figure S5: Eomes and Tbet expression in antigen-specific T cells (related to Figure 5): (A) HCV-specific T memory cells induced by virally vectored vaccine regimes (black; 8-26 weeks post boost vaccination) or by natural infection with CMV (grey) were identified by pentamer staining and split into T cell memory subsets using CD45RA and CCR7 staining (Naïve: CD45RA+CCR7+; Tcm: CD45RA-CCR7+; Tem: CD45RA-CCR7-; Temra: CD45RA+CCR7-). The co-expression of T-bet and Eomes was assessed on each T cell subset (CMV n=8; Ad/Ad vaccination n=8, Ad/MVA n=6). Line at median. (B) In four vaccinated individuals CMV-specific memory T cells could be detected and the expression pattern of Eomes and Tbet is shown in both vaccine induced HCV-specific T cells and CMV-specific T cells. **(C)** A comparison of the co-expression of T-bet and Eomes on vaccine induced T cells in individuals who received either ChAd3/Ad6 or ChAd3-MVA heterologous prime-boost vaccination regimes. Bars at median.

Supplementary Experimental Procedures

Intracellular staining and peptide stimulation

Peptide stimulation: 1x10^6 cells were stimulated for 2 hours at 37°C with either 10^{-4} M M38 or 10^{-4}M M45 peptide. As a positive control cells were stimulated with phorbol myristate acetate (PMA) (50ng/ml) and ionomycin (500ng/ml) or left untreated as a negative control. After 2 hours GolgiPlug (1μl/1ml final concentration) from BD Bioscience (Oxford, UK) was added to each well and cells incubated for a further 4 hours at 37°C. Cells were fixed with the cytofix/cytoperm solution from BD Bioscience (Oxford, UK) and stained and washed with Perm/Wash buffer from BD Bioscience (Oxford, UK).

Microarray data analysis

Gene expression data were obtained by hybridising a total of 12 samples from 4 experimental groups (n=3 per group) to Illumina MouseWG6 Expression BeadChips. Raw data were exported from the Illumina GenomeStudio software (v1.0.6) for further processing and analysis using R statistical software (v2.10) and BioConductor packages. Raw signal intensities were background corrected using array-specific measures of background intensity based on negative control probes, prior to being transformed and normalized using the 'vsn' package. Quality control analyses did not reveal any outlier samples. The dataset was then filtered to remove probes not detected (detection score <0.95) in any of the samples.

Statistical analysis was performed using the Linear Models for Microarray Analysis (limma) package. Differential expression between the experimental groups was assessed by generating relevant contrasts corresponding to the various two-group comparisons. Raw p-values were corrected for multiple testing using the false discovery rate controlling procedure of Benjamini and Hochberg, adjusted p-values below 0.01 were considered significant. Significant probe lists were then annotated using the relevant annotation file (MouseWG6_V2_0_R0_11278593_A) that was downloaded from the Illumina website (http://www.illumina.com) for further biological

investigation. To compare gene lists, these were ranked according to the t statistics or by log fold change and gene set enrichment analysis (GSEA) was run (www.broadinstitute.org/gsea/index.jsp). PCA of whole expression profiles was performed using R language and MeV software (http://www.tm4.org) using all genes probes, and repeated using the subset of significantly expressed transcription factors according ANOVA test across all samples groups.

Pentamer staining and flow cytometry

Fluorescence minus one (FMO) samples and isotype controls were performed using a CMV pentamer on CMV^{+} PBMCs, and fixed gating strategies were used throughout. The specificity of pentamers was tested on HLA-matched pre-vaccination samples from healthy volunteers (Barnes et al., 2012). PE-labeled class I pentamers loaded with HCV $NS3_{1406-1415}$ (KLSALGINAV), HCV $NS3_{1073-1081}$ (ATDALMTGY) CMV $pp65_{495-504}$ (NLVPMVATV), or FLU $M1/MP_{58–66}$ (GILGFVFTL) were obtained from ProImmune. The cells were co-stained with combinations of the following antibodies: CD3-PO (Invitrogen), CD8-Alexa700 (Biolegend), CCR7–PE-Cy7 (BD), CD45RA-FITC (BD; fluorescein isothiocyanate), T-bet-BV605 (Biolegend), Eomes-eFluor660 (eBiosciences).

PCA with CyTOF data

For PCA, cells were gated on live $CD3^{+}CD8^{+}$ T cells (principal component loaded using data from patient 319 at TW22 and then applied to the other data sets), and these events were exported to a tab-delimited text file with FlowJo v9.3.2 for further analysis with scripts written in MATLAB. FCS files containing these additional parameters were created using a custom algorithm written in Java (text to FCS script provided by W. Moore); pdb files were also created using MATLAB that could be read by PyMOL software (DeLano Scientific LLC). All MATLAB scripts started with transformation of data into logical biexponential scaling as described (Newell et al., 2012).

Cell preparation from bone marrow, lung and liver

Bone marrow was isolated by washing the femur shaft with PBS. Cells were passed through a 70µm nylon filter (BD) and red cell lysis was performed. Perfused livers were passed through a 70µm nylon filter (BD) and lymphocytes were purified by a Percoll (GE healthcare) gradient centrifugation. Lungs were minced with razor blades and incubated in PBS containing 60U/ml DNase (AppliChem) and 170U/ml collagenase II (Gibco) at 37°C for 45 min. Cell aggregates were dispersed by passing the digest through a 70µm nylon filter (BD). Absolute cell counts were determined by counting leukocytes in an improved Neubauer chamber.

RNA extraction and cDNA generation

Tetramer-sorted cells were resuspended in Trizol (Sigma-Aldrich, USA) and RNA was isolated by isopropanol precipitation, washed with ethanol 70% and resuspended in DEPC-water. RNA was DNAse treated with DNAse I (Invitrogen, Paisley, UK) and subjected to RT-PCR using 100ng purified RNA. For RT-PCR the high capacity cDNA archive Kit from Applied Biosystem (ABI PRISM, Warrington, United Kingdom) was used according to the specifications of the manufacturer.

Quantitative realtime PCR for selected genes

Quantitative real-time PCR was performed using a Light cycler 480 Real-Time PCR System (Roche Diagnostics) and the LightCycler 480 probes master reaction mix (Roche Diagnostics) following the manufacturer's protocol. Data analysis was performed with LightCycler 480 Software (Roche Diagnostics). Oligonucleotides were purchased from Eurofins MWG Operon (Ebersberg, Germany). Oligonucleotides sequences used as primers for quantitative real-time PCR and corresponding probes were designed according to the guidance of the Universal Probelibrary from Roche applied Science. Thermal cycling started with HotStarTaq activation during 10 min at 95°C. Thereafter 45 cycles of amplification were run consisting of 10 s at 95°C, 30 s 60°C, and 20 s of 72°C. A negative control, containing reagents only, and serial dilutions of cDNA were included in

each run. Each sample was measured as a triplicate and the average concentration was used. For LightCycler analysis, expression of hypoxanthine phosphoribosyltransferase gene (HPRT) was used for normalization. Relative expression of samples from naive and MCMV-infected or Ad-LacZ immunized mice was calculated by the comparative cycling threshold method ($\Delta\Delta C_T$).

Barnes, E., Folgori, A., Capone, S., Swadling, L., Aston, S., Kurioka, A., Meyer, J., Huddart, R., Smith, K., Townsend, R., *et al.* (2012). Novel adenovirus-based vaccines induce broad and sustained T cell responses to HCV in man. Sci Transl Med *4*, 115ra111.

Hertoghs, K.M., Moerland, P.D., van Stijn, A., Remmerswaal, E.B., Yong, S.L., van de Berg, P.J., van Ham, S.M., Baas, F., ten Berge, I.J., and van Lier, R.A. (2010). Molecular profiling of cytomegalovirus-induced human CD8+ T cell differentiation. The Journal of clinical investigation *120*, 4077-4090.

Newell, E.W., Sigal, N., Bendall, S.C., Nolan, G.P., and Davis, M.M. (2012). Cytometry by time-of-flight shows combinatorial cytokine expression and virus-specific cell niches within a continuum of CD8+ T cell phenotypes. Immunity *36*, 142-152.
